# Presenilin-1 controls glycolysis and identity of pancreatic beta cells

**DOI:** 10.1038/s42003-026-10581-6

**Published:** 2026-07-01

**Authors:** Zhanat Koshenov, Sandra Postic, Gabriela Schoiswohl, Savina van Amsterdam, Victoria Hois-Zelinka, Furkan E. Oflaz, Rene Rost, Oleksandra Tiapko, Benjamin Gottschalk, Martin Hirtl, Olaf G. Bachkoenig, Aigerim Koshenova, Yusuf C. Erdogan, Adlet Sagintayev, Anita Krnjic, Johannes U. Pfabe, Srdjan Sarikas, Bernhard Hochreiter, Juergen Gindlhuber, Matthias Schittmayer, Jelena Tadic, Barbara Ehall, Frank Madeo, Tobias Madl, Roland Malli, Ruth Birner-Gruenberger, Thomas Pieber, Tobias Eisenberg, Marjan Slak Rupnik, Wolfgang F. Graier

**Affiliations:** 1 Gottfried Schatz Research Center, Medical University of Graz, Graz, Austria; 2 Center for Physiology and Pharmacology/ Comprehensive Center for AI in Medicine, Medical University of Vienna, Vienna, Austria; 3 Division of Endocrinology and Diabetology, Department of Internal Medicine, Medical University of Graz, Graz, Austria; 4 Institute of Biotechnology and Biochemical Engineering, NAWI Graz, Graz University of Technology, Graz, Austria; 5 Institute of Science and Technology Austria - Imaging and Optics Facility, Klosterneuburg, Austria; 6 Institute of Chemical Technologies and Analytics, TU Wien, Vienna, Austria; 7 Institute of Molecular Biosciences, NAWI Graz, University of Graz, Graz, Austria; 8 BioTechMed-Graz, Graz, Austria; 9 Field of Excellence BioHealth, University of Graz, Graz, Austria; 10 Otto Loewi Research Center, Medical University of Graz, Graz, Austria; 11 Center of Medical Research, CF Bioimaging, Medical University of Graz, Graz, Austria; 12 Diagnostic and Research Institute of Pathology, Medical University of Graz, Graz, Austria; 13 Institute of Physiology, Faculty of Medicine, University of Maribor, Maribor, Slovenia

**Keywords:** Mechanisms of disease, Cell signalling

## Abstract

Presenilin-1 (PS1) is an endoplasmic reticulum protein, most known for its role in pathogenesis of familial Alzheimer’s Disease (AD). PS1 has been attributed roles in intracellular calcium homeostasis in the brain, as well as in the pancreatic beta cells, where it has been shown to be fundamental for the initial phase of glucose-induced insulin secretion. To gain mechanistic insight into beta cell autonomous function of PS1, we have examined various beta cell models and found that PS1 controls glycolytic flux of pancreatic beta cells by regulating sub-mitochondrial Ca^2+^ homeostasis. Transient knock down of PS1 yielded a glycolytic bottleneck at the step catalyzed by glyceraldehyde-3-phosphate dehydrogenase, resulting in defective glucose responsiveness. Moreover, we show that PS1 is needed for the preservation of beta cell identity, likely due to its role in glycolysis. Finally, we show that mouse islets from an in vivo model of AD mimic the defective glucose responsiveness seen in beta cells with transient PS1 knock down, while the identity loss and whole-body glucose homeostasis are not severely impacted, suggesting an adaptive response. Thus, the current work highlights beta cell autonomous role of PS1 and adds a layer of complexity in its impact on whole body metabolism.

## Abstract (web summary)

The work uncovered mechanistic details of presenilin-1-mediated control of beta cell metabolism and revealed a novel role of presenilin-1 as a regulator of beta cell identity.

## Introduction

Pancreatic beta cells function as metabolic sensors of the body. They detect and respond to elevated blood glucose by a series of metabolic events culminating in insulin secretion, which dictates whole-body glucose metabolism. The molecular machinery behind this robust response is tightly controlled, with Ca^2+^ ions playing the key regulatory roles^1^. Following glucose uptake by plasma membrane glucose transporters (mainly GLUT 2 in rodents and GLUT 1 and 3 in humans)^2^, glucose is metabolized by beta cells to generate ATP, increasing intracellular ATP concentration. The latter is implicated in closure of ATP-sensitive K^+^ channels, leading to membrane depolarization and entry of Ca^2+^ ions through voltage-gated Ca^2+^ channels. Resulting increase in cytosolic Ca^2+^ facilitates the fusion of insulin-containing vesicles with the cell membrane and release of insulin^3^. While most of the ATP is generated in the mitochondria, glycolysis plays a major role in beta cell metabolism^4^. Both mitochondrial oxidative phosphorylation (OXPHOS) and cytosolic glycolysis can be regulated by Ca^2+^ ions^1,5,6^, adding complexity to bioenergetic control of these highly specialized glucose sensor cells.

The molecular machinery of beta cells’ glucose metabolism is highly conserved and is part of beta cell identity^7^. At the genetic level, beta cell identity is maintained by activity of key transcription factors, such as pancreatic and duodenal homeobox 1 (PDX1), MAF BZIP Transcription Factor A (MafA), and NK6 homeobox 1 (NKX6.1)^7–10^. Loss of beta cell identity is associated with the development of types 1 and 2 diabetes^8,9,11,12^. Interestingly, metabolic control of cell fate and identity has recently emerged as a prominent topic across many fields of biology. Thus, identification of novel genetic and metabolic control mechanisms of metabolism and identity of beta cells can have a fundamental impact on our understanding of beta cell function and can provide new therapeutic targets against diabetes.

There is a large body of literature associating Alzheimer’s disease (AD) with diabetes and often calling AD a diabetes of the brain^13–16^. Indeed, there are striking similarities between the diseases, especially when it comes to glucose metabolism and Ca^2+^ signaling^13,17^. Our previous work has shown that presenilin-1 (PS1) plays a fundamental role in establishing an endoplasmic reticulum (ER) Ca^2+^ leak directed towards mitochondria that is needed for proper glucose responsiveness of beta cells^18,19^. This molecular function of PS1 in pancreatic beta cells displays parallels in neurons, where it is also responsible for ER Ca^2+^ leak. Loss or mutation of PS1 leads to neurodegenerative disorders, including familial AD^17^. We undertook the current study to investigate whether PS1 could provide a mechanistic link between the two devastating disorders of the brain and pancreas. Since more is known about the role of PS1 in the brain, we focused on the impact of loss or mutation of PS1 in the pancreas to identify its detailed mechanism of action in pancreatic beta cells. As a result, we uncovered mechanistic details of PS1-mediated control of beta cell metabolism and revealed a role of PS1 as a regulator of beta cell identity.

## Results

### Presenilin-1 is important for glucose metabolism preceding mitochondrial entry of pyruvate in pancreatic beta cells

**Fig. 1:**
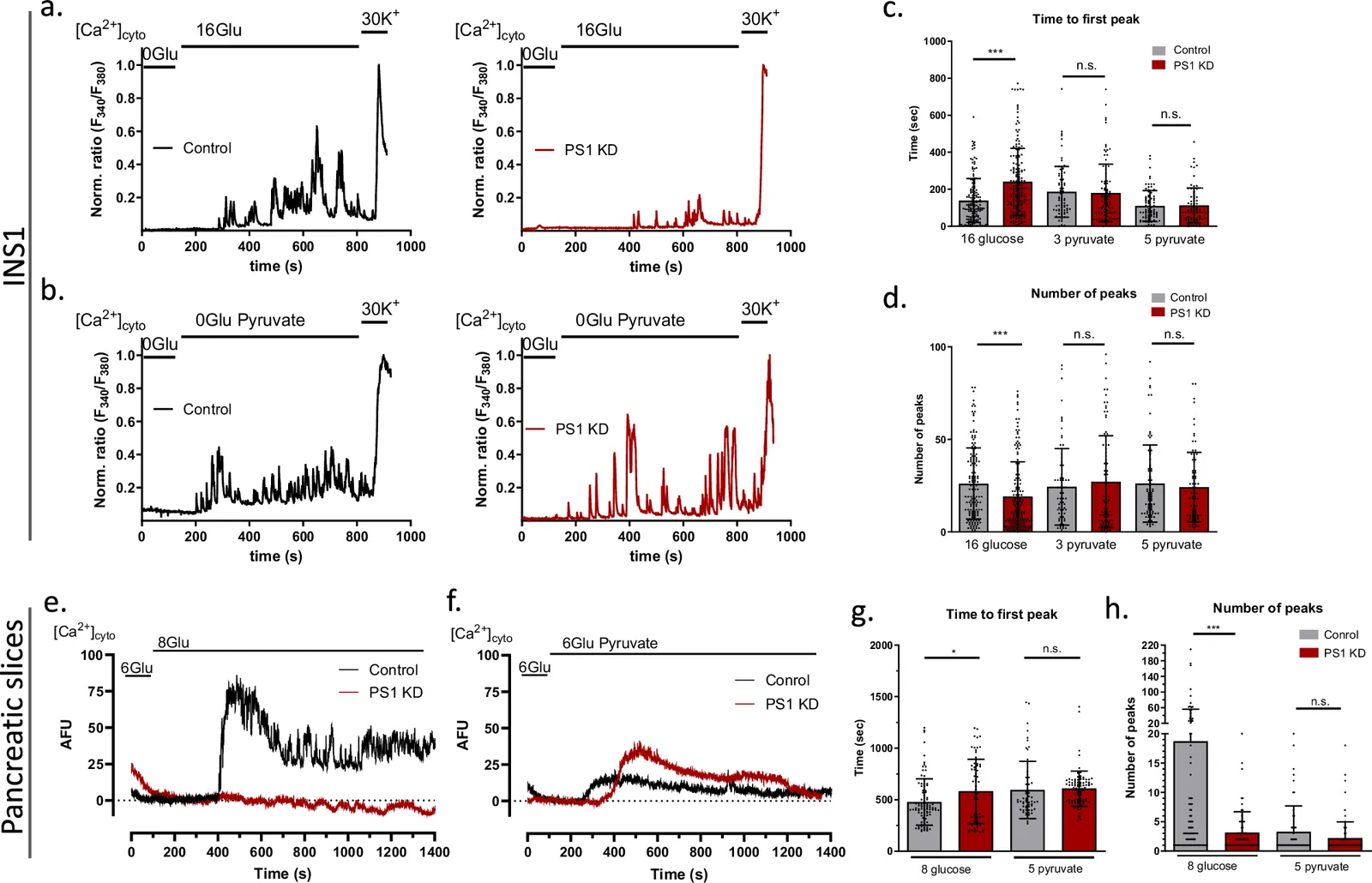
PS1 is important for glucose metabolism preceding mitochondrial entry of pyruvate. **a** Representative FURA-2 AM traces showing cytosolic Ca^2+^ oscillations in response to elevation of extracellular glucose in control (left, black) and PS1 KD (right, red) INS1 cells. **b** Representative traces showing cytosolic Ca^2+^ oscillations in response to elevation of extracellular pyruvate in control (left, black) and PS1 KD (right, red) INS1 cells. **c** Statistical analysis of experiments as shown in (**a**) and (**b**), bar graphs (+/-SD) show a time delay between the addition of 16 mM glucose, 3 mM or 5 mM pyruvate, and the onset of Ca^2+^ oscillations. **d** Bar graphs (+/-SD) show the number of cytosolic Ca^2+^ peaks for experiments as shown in (**a**) and (**b**) (n for (**c**) and (**d**)**:** control 16 glucose, *n* = 157, PS1 KD 16 glucose 167, control 3 pyruvate, *n* = 78, PS1 KD 3 pyruvate, *n* = 93, control 5 pyruvate, *n* = 81, PS1 KD 5 pyruvate, *n* = 82). **e** Representative traces showing cytosolic Ca^2+^ oscillations in response to elevation of extracellular glucose in control (black) and PS1 KD (red) beta cells in pancreatic organ slices. **f** Representative traces showing cytosolic Ca^2+^ oscillations in response to elevation of extracellular pyruvate in control (black) and PS1 KD (red) beta cells in pancreatic organ slices. **g** Statistical analysis of experiments as shown in (**e**) and (**f**), done on pancreatic organ slices, bar graphs (+/-SD) show a time delay between the addition of 8 mM glucose or 5 mM pyruvate, and the onset of Ca^2+^ oscillations. **h** Bar graphs (+/-SD) show the number of cytosolic Ca^2+^ peaks in pancreatic organ slices in response to glucose or pyruvate stimulation (n for (**g**) and (**h**)**:** control 8 glucose, *n* = 164, PS1 KD 8 glucose 148, control 5 pyruvate, *n* = 137, PS1 KD 5 pyruvate, *n* = 172). Unpaired t-test, n.s. *p* > 0.05, * *p* < 0.05, ** *p* < 0.01, *** *p* < 0.001.

We have previously shown that PS1 establishes an ER Ca^2+^ leak in pancreatic beta cells that is important for mitochondrial respiration and glucose-induced insulin secretion^19^. To dissect the metabolic role of PS1, we evaluated glucose- and pyruvate-induced Ca^2+^ oscillations in control and PS1 knockdown (KD) rat pancreatic beta cell line (INS1), and primary mouse pancreatic organ slices (Fig. 1, Supplementary Fig. 1a, b). In line with our previous work^19^, PS1 KD showed delayed and reduced number of Ca^2+^ oscillations when stimulated with glucose. Interestingly, stimulating the beta cells with pyruvate, a mitochondrial fuel, showed no difference between control and PS1 KD beta cells, suggesting that mitochondrial metabolism of beta cells lacking PS1 is not defective. This claim was further supported by direct measurement of mitochondrial ATP production in control and PS1 KD beta cells when stimulated with glucose or pyruvate (Supplementary Fig. 1c–f). While basal ATP and glucose-stimulated ATP production were lower in PS1 KD cells, pyruvate stimulation resulted in the same magnitude of ATP production in control and PS1 KD beta cells. Since decreased glucose uptake may explain the observed phenotypes under glucose stimulation experiments, we tested the impact of PS1 KD on glucose uptake and found that it did not reduce it, but rather resulted in a slightly increased plateau phase following glucose uptake (Supplementary Fig. 1g, h). Overall, these findings suggest that PS1 contributes to beta cell function by having a role in glucose metabolism following glucose uptake and preceding pyruvate entry into mitochondria.

### Presenilin-1 is needed for glycolytic flux of pancreatic beta cells

**Fig. 2:**
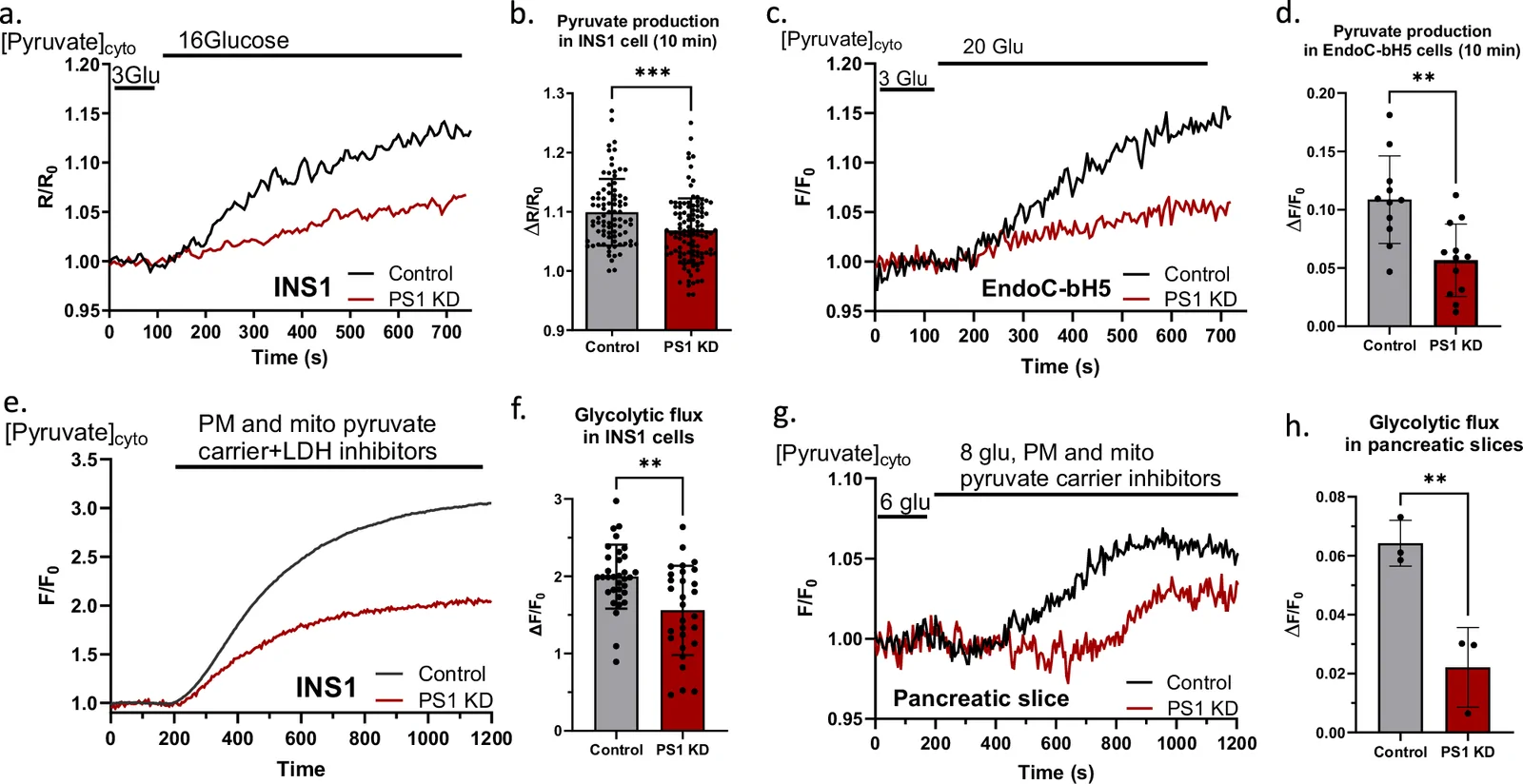
Presenilin-1 controls glycolytic flux in pancreatic beta cells. **a** Representative Pyronic traces depicting cytosolic pyruvate dynamics in response to increased extracellular glucose in control (black) and PS1 KD (red) INS-1 cells. **b** Statistical analysis of the experiment shown in **a**, bar graphs (+/-SD) show change in cytosolic pyruvate after 10 min in 16 mM glucose (control, *n* = 85, PS1 KD, *n* = 112). **c** Representative PyronicSF traces depicting cytosolic pyruvate dynamics in response to increased extracellular glucose in control (black) and PS1 KD (red) EndoC-bH5 cells. **d** Statistical analysis of the experiment shown in **c**, bar graphs (+/-SD) show change in cytosolic pyruvate after 10 min in 20 mM glucose (control, *n* = 11, PS1 KD, *n* = 12). **e** Representative PyronicSF traces depicting glycolytic flux experiment in control (black) and PS1 KD (red) INS-1 cells. **f** Quantification of the basal glycolytic flux (±SD) as a change in cytosolic pyruvate after 15 min perfusion with the inhibitor mix (control, *n* = 33, PS1 KD, *n* = 29). **g** Representative PyronicSF traces in response to increased extracellular glucose and application of pyruvate carrier inhibitors in control (black) and PS1 KD (red) beta cells in primary pancreatic slices. **h** Quantification of the stimulated glycolytic flux (+/-SD) as a change in cytosolic pyruvate after 15 min in 8 mM glucose and inhibitor mix (control, *n* = 3, PS1 KD, n = 3). Unpaired t-test, n.s. *p* > 0.05, * *p* < 0.05, ** *p* < 0.01, *** *p* < 0.001.

In order to test if PS1 plays a role in glycolytic activity, we measured cytosolic pyruvate production after the elevation of extracellular glucose in INS-1 cells using pyruvate biosensors^20,21^. PS1 KD did not affect basal pyruvate level at low glucose (3 mM) (Supplementary Fig. 2a), but it reduced pyruvate production upon glucose stimulation in INS1 and human cultured beta cells (EndoC-bH5)^22^ (Fig. 2a–d), suggesting that PS1 plays a role in beta cell glycolysis upon glucose stimulation.

Additionally, we measured basal glycolytic flux at fixed glucose concentration by acute inhibition of pyruvate exit routes by inhibiting mitochondrial pyruvate carrier (MPC) using 10 µM UK-5099^23^, pyruvate and lactate extrusion by monocarboxylate transporter (MCT) using 1 µM AR-C155858^24^, and lactate dehydrogenase (LDH) using 1 µM GSK-2837808A^25^. The resulting accumulation of pyruvate upon inhibiting its exit routes was assessed as steady-state glycolytic flux. PS1 KD reduced the steady-state glycolytic flux in INS-1 cells by about 50% (Fig. 2e, f). These experiments showed that PS1 indeed has a role in glycolytic flux, which could explain the reason for reduced glucose sensitivity of beta cells and islets lacking PS1 (Fig. 1). To further corroborate these findings, we measured glycolytic activity in mouse pancreatic organ slices with transient PS1 KD. A combination of increased extracellular glucose with PM and mitochondrial pyruvate carrier inhibition yielded pyruvate accumulation that was reduced by PS1 KD in primary pancreatic organ slices (Fig. 2g, h).

Furthermore, to supplement the imaging-based readouts, we have performed an NMR-based metabolic flux assay in INS1 cells upon glucose stimulation. The NMR analysis showed lactate accumulation in INS1 cells upon glucose stimulation, which was significantly reduced in PS1 KD cells, while the LDHa levels were unchanged (Supplementary Fig. 2b, c), suggesting that hampered lactate production is not due to a change in LDHa expression, but likely upstream of it. Citrate, a TCA cycle intermediate, on the other hand, showed a decrease in PS1 KD cells upon glucose stimulation, while control cells maintained a more or less stable levels (Supplementary Fig. 2d), hinting at reduced supply of intermediates to TCA cycle in the absence of PS1.

Overall, experiments done on all three beta cell models using different techniques support the notion that PS1 plays a role in beta cell glycolysis.

### Presenilin-1 controls beta cell glycolysis by regulating malate-aspartate shuttle activity

We and others have shown that a reduction in mitochondrial intermembrane space (IMS) Ca^2+^ level can inhibit the activity of IMS residing Ca^2+^ sensitive NADH shuttles, which in turn can influence glycolysis^5,6^. As we have previously shown that PS1 is responsible for mitochondria-directed ER Ca^2+^ leak^18^, we wondered if the IMS Ca^2+^ level is affected by PS1. Thus, we assessed steady-state IMS Ca^2+^ level using genetically encoded biosensor targeted to IMS^26^ and found that PS1 KD resulted in reduced basal IMS Ca^2+^ in INS1 cells (Supplementary Fig. 3a), which opened a new inquiry into the mechanism of PS1-dependent control of beta cell glycolysis. Of note, we found slightly increased ER Ca^2+^ level in PS1 KD cells, in line with reduced ER Ca^2+^ leak, but unchanged cytosolic Ca^2+^ (Supplementary Fig. 3b, c), which indicated that the PS1 mediated ER Ca^2+^ leak is directed towards mitochondria. As ER Ca^2+^ overload can be an indicator of ER stress, we assessed ER stress by evaluating X-box binding protein 1 (XBP1) splicing^27^. We found no difference in XBP1 splicing between control and PS1 KD INS1 cells (Supplementary Fig. 3d), suggesting the slight increase in ER Ca^2+^ content is not causing or caused by ER stress.

**Fig. 3:**
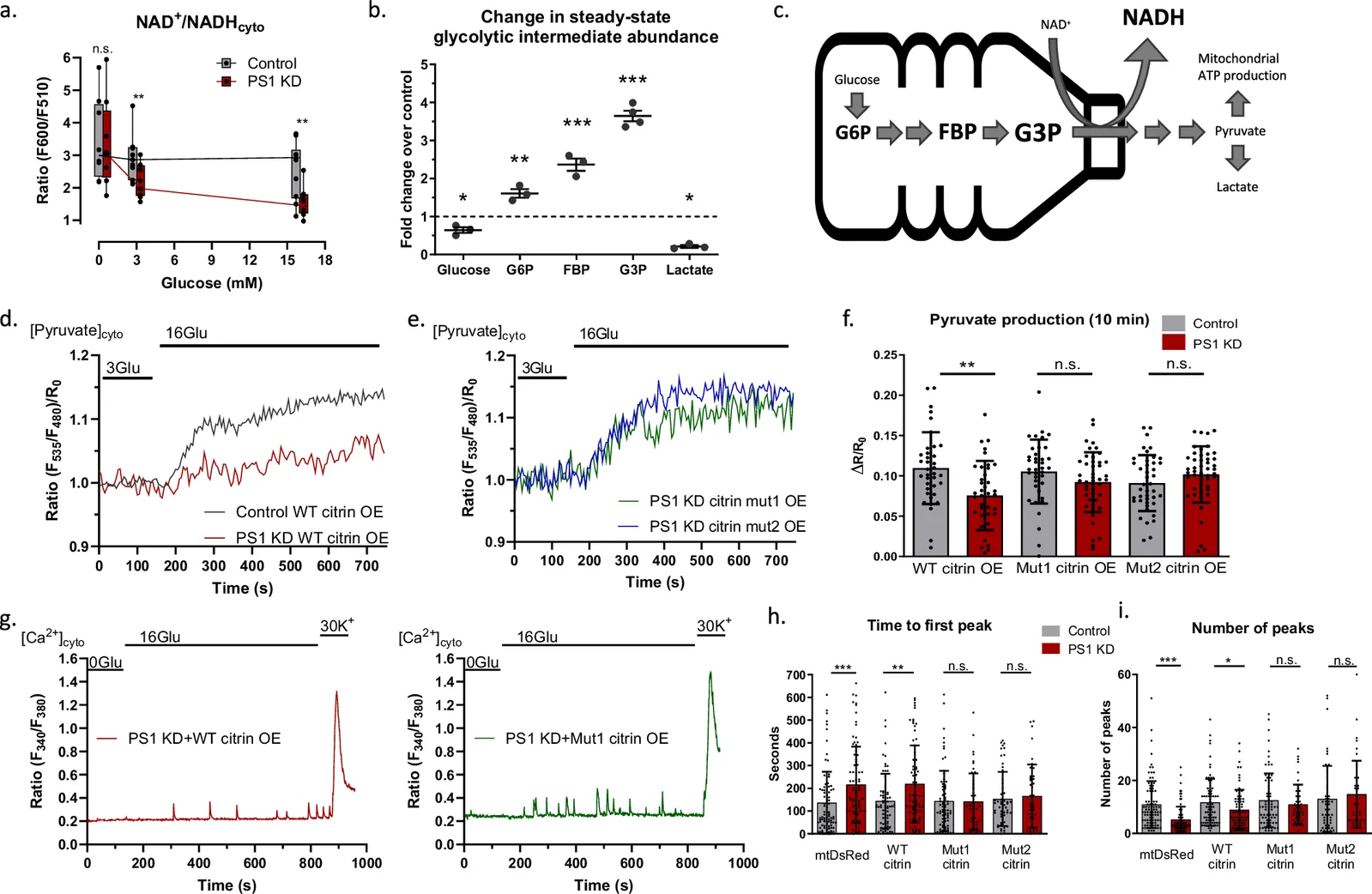
Presenilin-1 controls the glycolytic flux by regulating malate-aspartate shuttle activity. **a** Glucose dependent changes in the cytosolic NAD^+^/NADH ratio is shown as the Peredox biosensor emission ratio for control (black) and PS1 KD (red) INS-1 cells (box plot shows the median and min to max distribution; control: 0 glucose *n* = 8, 3 glucose *n* = 11, 16 glucose *n* = 10; PS1 KD: 0 glucose *n* = 8, 3 glucose *n* = 11, 16 glucose *n* = 10). **b** Statistical analysis of mass spectrometry assay of glycolytic intermediates in INS-1 cells; metabolite abundance of PS1 KD cells is normalized to control (*n* = 3-4, error bars are SD). **c** Graphical representation of the hypothesized glycolytic bottleneck in PS1 KD beta cells; font size represents metabolite abundance. **d** Representative cytosolic pyruvate traces in response to increased extracellular glucose in control (black) and PS1 KD (red) INS-1 cells overexpressing wild-type citrin construct. **e** Representative cytosolic pyruvate traces in response to increased extracellular glucose in PS1 KD INS-1 cells overexpressing wild mutant citrin constructs. **f** Statistical analysis of the experiments shown in (**d**) and (**e**), bar graphs (±SD) show changes in cytosolic pyruvate after 10 min in 16 mM glucose (control: WT citrin OE *n* = 37, mut1 citrin OE *n* = 38, mut2 citrin OE *n* = 42; PS1 KD: WT citrin OE *n* = 38, mut1 citrin OE *n* = 45, mut2 citrin OE n = 46). **g** representative FURA-2 AM traces showing cytosolic Ca^2+^ oscillations in response to elevation of extracellular glucose in PS1 KD + WT citrin OE (left, red) and PS1 KD + Mutant 1 citrin OE (right, green) INS1 cells. **h** Statistical analysis of experiments shown in (**g**), bar graphs (+/-SD) show a time delay between the addition of 16 mM glucose and the onset of Ca^2+^ oscillations. **i** Bar graphs (+/-SDF) show the number of cytosolic Ca^2+^ peaks for experiments shown in (**g**) (n for (**h**) and **i:** controls: mtDsRed OE *n* = 92, WT citrin OE *n* = 79, mut1 citrin OE *n* = 75, mut2 citrin OE *n* = 53; PS1 KD: mtDsRed OE *n* = 84, WT citrin OE *n* = 74, mut1 citrin OE *n* = 46, mut2 citrin OE *n* = 41).

To test if reduced Ca^2+^ homeostasis at mitochondrial IMS could have impaired NADH shuttle activity, we measured the cytosolic NAD^+^/NADH ratio in INS-1 cells using genetically encoded biosensor Peredox^28^. PS1 KD resulted in a glucose-dependent reduction of cytosolic NAD^+^/NADH ratio (Fig. 3a), suggesting an inability of these cells to recycle cytosolic NADH back to NAD^+^. In support of these results, NMR analysis showed reduced level of total NAD^+^ in INS1 cells with PS1 KD (Supplementary Fig. 3e). As this metabolic defect may be the reason behind the hampered glycolytic flux caused by PS1 KD, we hypothesized that PS1 controls glycolysis by maintaining IMS Ca^2+^ homeostasis and supporting the activity of Ca^2+^-sensitive NADH shuttles in the IMS. These shuttles recycle cytosolic NADH back to NAD^+^, supplying NAD^+^ to maintain the activity of glyceraldehyde-3-phosphate dehydrogenase (GAPDH). Accordingly, we hypothesized that a bottleneck would form within glycolysis at the GAPDH-catalyzed step in PS1 KD cells.

To test our hypothesis, we measured glycolytic intermediates using mass spectrometry. Our analysis revealed accumulation of metabolites between glucose 6-phosphate (G6P) and glyceraldehyde 3-phosphate (G3P), and the reduction of lactate in PS1 KD cells (Fig. 3 b), supporting our bottleneck hypothesis (Fig. 3c). Using the fructose 1,6-bisphosphate (FBP) biosensor, Hylight^29^, we could confirm increased FBP levels in the cytosol of PS1 KD INS1 cells (Supplementary Fig. 4f). Curiously, the mass spectrometry analysis also showed decreased glucose level in PS1 KD cells. Since no defects in glucose uptake upon PS1 KD were found (Supplementary Fig. 1g, h), we assumed that it could be an adaptive mechanism in response to hampered glycolytic flux. No glycolytic intermediates between G3P and lactate were detectable, likely due to low abundance, which can also explain the fact that we observed no significant difference in basal pyruvate level between control and PS1 KD cells in 3 mM glucose (Supplementary Fig. 2a). Additionally, NMR analysis showed reduced aspartate level in PS1 KD cells (Supplementary Fig. 3g), which has been shown to be a consequence of hampered MAS activity^30^.

In order to challenge our proposed mechanism of PS1-mediated control of glycolysis in a more direct manner, we attempted to rescue the glycolysis by manipulating the malate-aspartate shuttle (MAS). MAS is the main NADH shuttle in pancreatic beta cells, which expresses aspartate-glutamate mitochondrial carrier 1 (AGC1, or aralar), as the Ca^2+^ sensitive component^31–33^. AGC1 is half-maximally stimulated at around 300 nM Ca2+^33^, and thus we wondered if we could rescue glycolysis in PS1 KD cells by overexpressing AGC2 (citrin), a homolog of AGC1 in non-excitable cells, which is active at lower Ca^2+^ concentrations^33^. Using pyruvate production upon glucose elevation in INS-1 cells as a readout, we found that AGC2 overexpression (OE) did not restore pyruvate production of PS1 KD cells (Fig. 3d), suggesting that the activity of the wild-type AGC2 might still be affected by the reduction of IMS Ca^2+^ level. Consequently, we generated AGC2 mutants that potentially mimic its conformational change upon Ca^2+^ binding. For that, we relied on available AGC1-2 crystal structures^34^ and introduced single amino acid exchanges at glutamine 67 (mutant 1) or glutamine 76 (mutant 2) to lysine. OE of either of these mutants in PS1 KD cells ultimately rescued their pyruvate production upon glucose stimulation (Fig. 3e, f, Supplementary Fig. 3h), supporting our hypothesis on PS1-mediated control of glycolytic flux through modulation of Ca^2+^-sensitive MAS activity. Furthermore, OE of Ca^2+^ bound state mimicking AGC2 mutants, but not the wild-type (WT) AGC2, rescued glucose-stimulated Ca^2+^ oscillations in PS1 KD INS-1 cells (Fig. 3g–i), further confirming the mechanism of PS1-mediated control of beta cell glucose metabolism through modulation of MAS activity.

### Presenilin-1 preserves beta cell identity by maintaining glycolytic flux

**Fig. 4:**
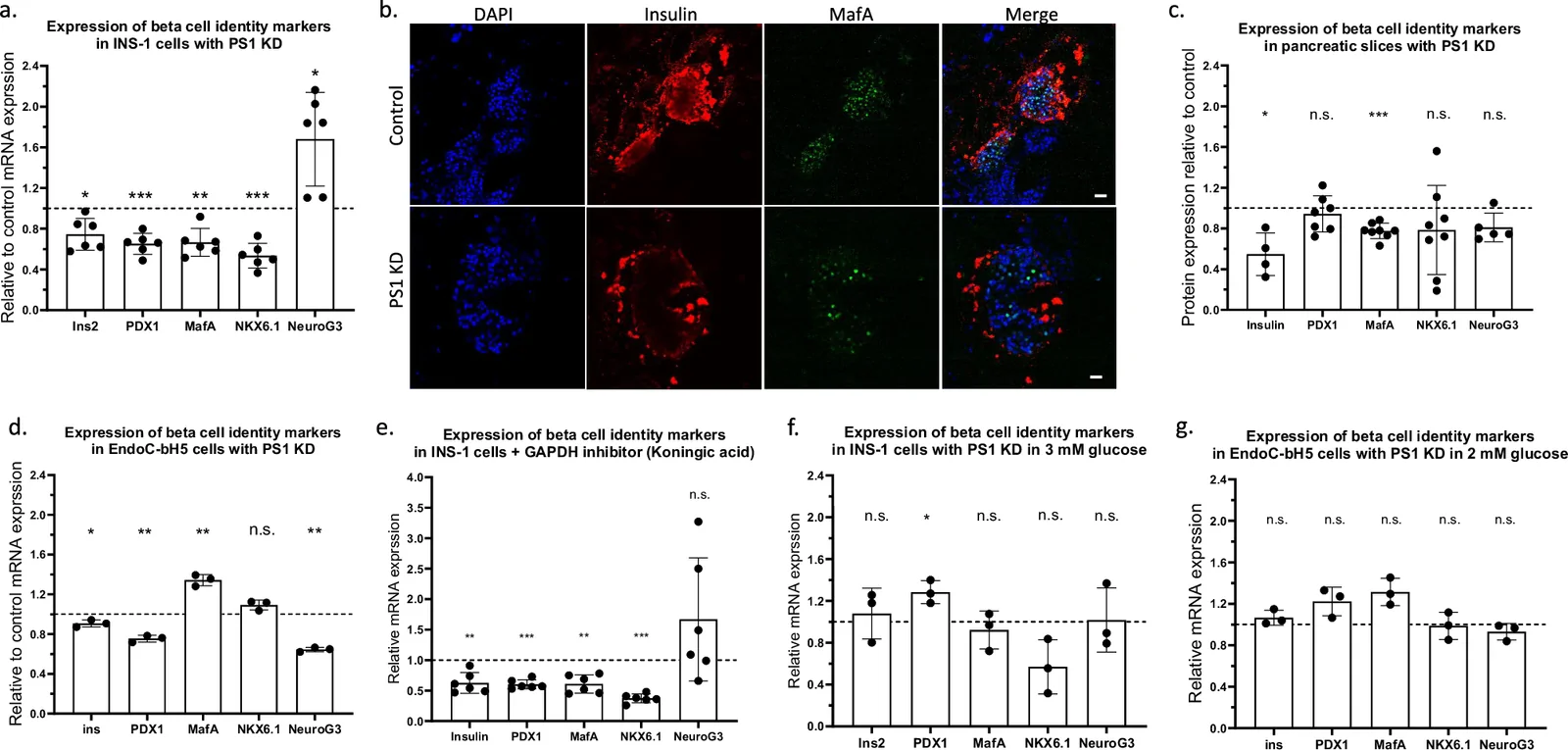
Presenilin-1 preserves beta cell identity. **a** Bar graphs show mean (±SD) mRNA expression of insulin, PDX1, MafA, NKX6.1 and Neurogenin-3 in PS1 KD INS-1 cells, normalized to respective mRNA expression in control cells (dotted line at Y = 1; *n* = 6). **b** Representative immunofluorescence images of primary pancreatic slices stained with anti-insulin (red) and anti MafA (green) antibodies (red), scale bar is 20 µm. **c** Statistical analysis (mean +/-SD) of immunofluorescence images taken from control and PS1 KD primary pancreatic slices stained with antibodies against insulin, PDX1, MafA, NKX6.1 and Nerogenin-3 (*n* = 4–8). **d** Bar graphs show mean (±SD) mRNA expression of insulin, PDX1, MafA, NKX6.1 and Neurogenin-3 in PS1 KD EndoC-bH5 cells, normalized to respective mRNA expression in control cells (dotted line at Y = 1; *n* = 3). **e** Bar graphs show mean (+/-SD) mRNA expression of beta cell identity markers in INS1 cells supplemented with GAPDH inhibitor (5 µM) for 48 hours prior to the analysis; values are normalized to DMSO treated controls, *n* = 6. **f**,**g** Bar graphs show mean (±SD) mRNA expression of beta cell identity markers in PS1 KD INS-1 (**f**) and EndoC-bH5 (**g**) cells grown under low glucose condition for 48 h prior to the analysis; values are normalized to control expression under low glucose condition, *n* = 3. Unpaired t-test (**c**), paired t-test (**a**,**d–g**), n.s. *p* > 0.05, * *p* < 0.05, ** *p* < 0.01, *** *p* < 0.001.

Glucose metabolism plays a central role in the identity of pancreatic beta cells^7^. As our experiments indicate an involvement of PS1 in glucose responsiveness of beta cells, we wondered if it also influences beta cell identity. For that, we analyzed rat and human pancreatic beta cells (INS1 and EndoC-bH5, respectively), as well as mouse islets, for beta cell identity markers and found that beta cells with PS1 KD had altered expression of mature beta cell genes, indicative of identity loss (Fig. 4a–d, Supplementary Fig. 4a–c). Interestingly, the pattern of changes in marker expression was different among the three tested models. In INS1 cells, PS1 KD resulted in a reduction of all tested mature beta cell marker genes, such as insulin (*ins2*), *PDX1, MafA*, and *NKX6.1*, while the expression of the progenitor cell marker Neurogenin-3 (*NeuroG3*) was increased (Fig. 4a), suggesting a developmental step-back towards a progenitor-like cell type. In human EndoC-bH5 beta cells, PS1 KD changed the expression of some of the beta cell identity markers, with insulin, *PDX1*, and *NeuroG3* being significantly reduced, while *MafA* expression was increased (Fig. 4d). Reduction in insulin and *PDX1* clearly shows a dedifferentiation of EndoC-bH5 beta cells, as does a dramatic loss of *NeuroG3*, which might suggest an even harsher loss of identity. PS1 KD in primary mouse pancreatic organ slices also reduced insulin and *MafA* expression (Fig. 4b, c). Although there is a variation in the expression pattern among the three used beta cell models, the overall changes support the assumption of a loss of beta cell identity by downregulation of PS1, identifying a previously unknown role for PS1 in maintaining beta cell identity.

To test whether it is the role of PS1 in glucose metabolism that contributes to the preservation of beta cell identity, we have attempted several manipulations to rescue or mimic the loss of identity caused by PS1 KD. First, we provided beta cells with extra pyruvate, which rescued Ca^2+^ oscillations and ATP production (Fig. 1, Supplementary Fig. 1c–f). However, extra pyruvate did not rescue the loss of beta cell identity (Supplementary Fig. 4d), suggesting that the glycolytic defect and the bottleneck caused by PS1 KD, but not reduced mitochondrial fuel supply, might be the reason for the loss of identity. In order to mimic the effect of PS1 KD on glycolysis, we used koningic acid (KA), an inhibitor of GAPDH^35^. 5 µM KA application triggered loss of identity in INS1 cells that closely mimicked the pattern seen in PS1 KD cells (Fig. 4e), suggesting an overlapping mechanism for the loss of identity. As inhibition of GAPDH by KA or an indirect inhibition by reduction in NAD^+^/NADH ratio caused by PS1 KD would result in reduced glycolysis (Fig. 2a–h) and in formation of the glycolytic bottleneck^36^ (Fig. 3a–c), we wanted to understand which of these two phenotypes is responsible for the loss of identity. To address this question, we reasoned that reduction of culture glucose should delineate the effects of reduced glycolysis and the glycolytic bottleneck, as it would mimic the former and prevent the latter. Interestingly, lowering culture glucose (3 mM for INS1, 2 mM for EndoC-bH5) preserved the beta cell identity markers in both models with PS1 KD (Fig. 4f, g), suggesting that the glycolytic bottleneck was responsible for the loss of beta cell identity.

Recent studies have shown that glycolytic intermediates upstream of phosphofructokinase and downstream of GAPDH can cause defects in beta cell function by upregulating mTORC1 activity^36,37^. In line with these studies and the glycolytic bottleneck we observed, PS1 KD resulted in elevation of mTORC1 activity in INS1 cells (Supplementary Fig. 4e). Application of rapamycin, an mTORC1 inhibitor, partially alleviated the loss of identity in PS1 KD beta cells (Supplementary Fig. 4f, g), hinting that mTORC1 could possibly be involved downstream of PS1 in preserving beta cell identity. In summary, we have revealed that PS1 preserves beta cell identity by maintaining smooth glycolytic flux, which, if disturbed, can cause loss of identity.

### Genetic drivers of the Familial Alzheimer’s Disease cause impaired glucose responsiveness in pancreatic islets and hint at the onset of identity loss

**Fig. 5:**
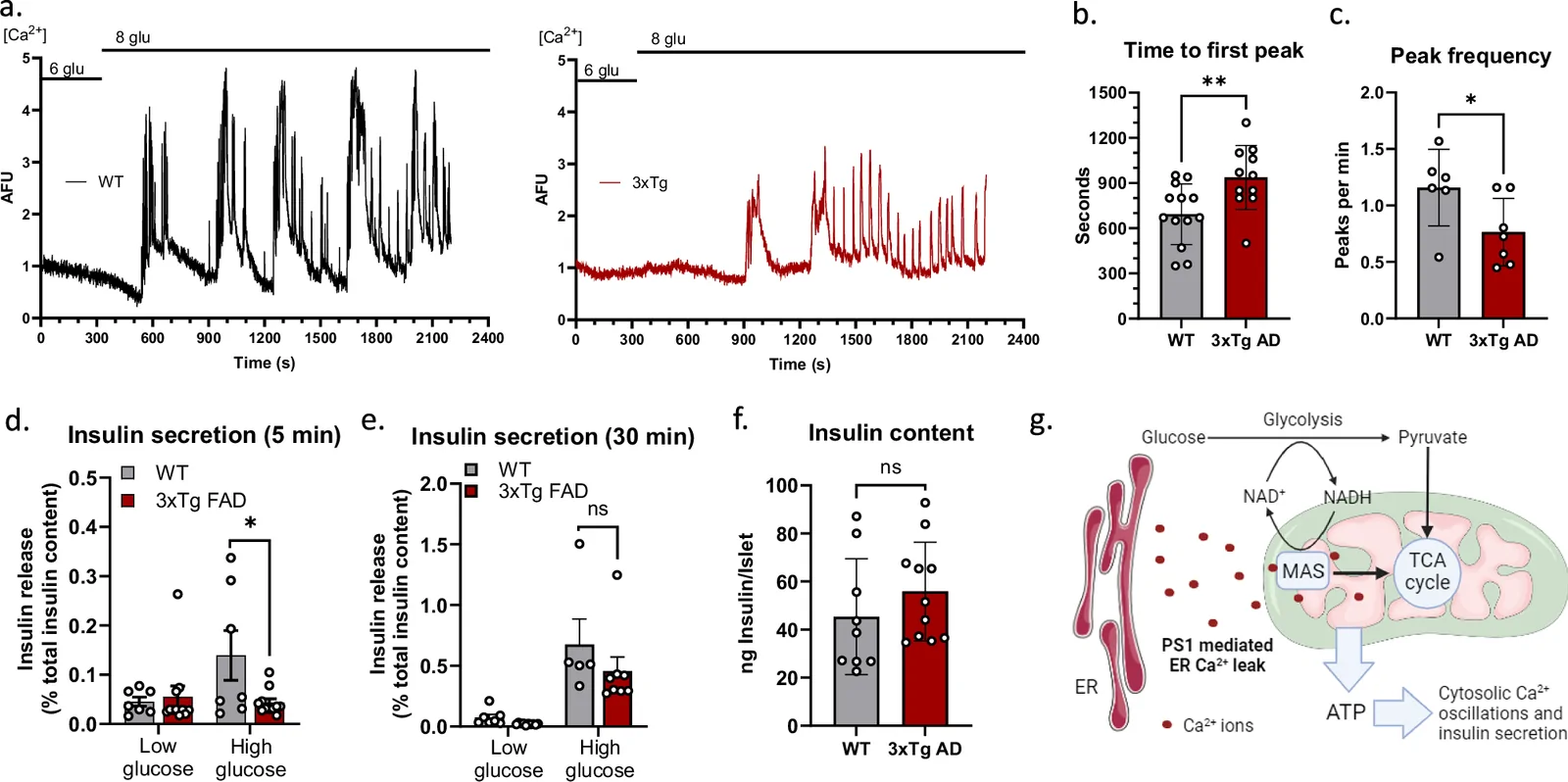
Genetic drivers of the Familial Alzheimer’s Disease cause impaired glucose responsiveness in pancreatic islets. **a** Representative traces showing cytosolic Ca^2+^ oscillations in response to elevation of extracellular glucose in beta cells from pancreatic slices of WT (left) and 3xTg AD model (right) animals. **b** Statistical analysis of experiments as shown in **a**, bar graphs (+/-SD) show a time delay between the addition of 8 mM glucose and the onset of Ca^2+^ oscillations (*n* = 11–13). **c** Bar graphs (±SD) show the cytosolic Ca^2+^ peak frequency for experiments as shown in (**a**) (*n* = 6-7). **d** Insulin secretion (+/- SD) during the first 5 min of glucose stimulation by pancreatic islets from WT and 3xTg AD model mice (*n* = 7–11). **e** Total insulin content (± SD) in islets from WT and 3xTg FAD mice (*n* = 7–11). **f** Extended insulin secretion (±SD) during the first 30 minutes of glucose stimulation by pancreatic islets from WT and 3xTg AD model mice (*n* = 7–11). **g** Graphical representation of the PS1-mediated control of beta cell metabolism. Unpaired t-test, n.s. *p* > 0.05, * *p* < 0.05, ** *p* < 0.01.

Since PS1 mutations are a major cause of familial AD^17,38^ and given the large amount of published work claiming high correlation between Alzheimer’s disease and diabetes^13–16,39^, we wondered if drivers of AD could cause dysfunction in pancreatic beta cells, similar to what we have observed with transient KD of PS1. Hence, we have used a triple transgenic (3xTg) AD mouse model that has a brain-restricted expression of human amyloid precursor protein with the Swedish mutation (APPSwe) and tubulin-associated unit (Tau) with P301L mutation, while the human PS1 gene with M146V mutation, which lacks the Ca^2+^ leak function^17^, is expressed in the whole body, including the pancreas^40,41^. Glucose-stimulated Ca^2+^ oscillations were delayed and reduced in pancreatic organ slices from 3xTg mice (Fig. 5a–c), similar to our current findings with transient PS1 KD (Fig. 1). In line with the delayed onset of glucose-stimulated Ca^2+^ oscillations, islets from the AD model mice showed decreased insulin secretion during the first 5 min of glucose stimulation (Fig. 5d). There was no difference in total islet insulin content or insulin secretion measured over a prolonged time (30 min) (Fig. 5e, f), which is similar to our previous findings with PS1 KD in pancreatic islets^19^. These results, paired with our mechanistic insight on the role of PS1 in glycolytic flux, emphasize the importance of functional PS1 in glucose responsiveness of pancreatic beta cells (Fig. 5g) and bring forth a potential mechanistic link between AD and pancreatic dysfunction.

Interestingly, despite the reduction of the initial phase of insulin release, there was only a minimal change in the blood glucose level of *ad libitum* fed AD model mice (Supplementary Fig. 5a), likely due to no change in later stages of insulin release. Furthermore, we analyzed islets from 3xTg mice for markers of mature beta cells and found that AD model mice had slightly reduced expression of *Pdx1*, *MafA*, and *Nkx6.1*, while the expression of insulin and *NeuroG3* was unaffected (Supplementary Fig. 5b). Overall, similarities between the glucose responsiveness of islets from the in vivo AD mouse model and transient PS1 KD emphasize the importance of PS1 for glucose metabolism in pancreatic beta cells. While the loss of identity in transient KD models is more pronounced, the in vivo model might have additional compensatory mechanisms in place and requires further investigation.

## Discussion

The role of the pancreas as a driver of bodily metabolism is dependent on the ability of its resident cells to detect and respond to certain metabolic cues. Pancreatic beta cells detect and respond to an elevation in blood glucose level by releasing insulin, thus steering the whole body glucose metabolism. Inability of the beta cells to properly respond to elevations of blood glucose can lead to drastic metabolic phenotypes, including dysfunction of the CNS^42,43^. Our previous work on the function of PS1 in pancreatic beta cells left open questions regarding the detailed mechanism of PS1-mediated control of mitochondrial bioenergetics, as well as the involvement of PS1 in glucose responsiveness of beta cells^18,19^. To address these questions, we have used four different models of pancreatic beta cells: rat INS-1 insulinoma cell line, recently developed EndoC-bH5 human beta cell line^22^, primary mouse pancreatic islets, and pancreatic organ slices from WT and triple transgenic in-vivo knock-in mouse model of Alzheimer’s disease (3xTg AD). To decipher the intricate control mechanisms of beta cell metabolism and the role of PS1, we utilized recent advances in live-cell imaging using various ion and metabolite biosensors, where the INS-1 cell line offered the most flexibility in terms of expression and robustness. Our previous work demonstrated that the results obtained using the INS-1 rat insulinoma cell line were comparable to the results from freshly isolated mouse pancreatic islets^17,19^. As a result, the INS-1 cell line was used for most of the experiments, while the human EndoC-bH5 beta cell line and primary mouse islets and organ slices were used to confirm key findings.

Using the aforementioned models of beta cells with transient KD of PS1, we could show that bypassing glycolysis and directly feeding beta cells with pyruvate rescues mitochondrial ATP production and cytosolic Ca^2+^ oscillations in PS1 KD cells. There were, however, differences between the INS1 cell line and primary pancreatic organ slices in their response to pyruvate stimulation (Fig. 1). It has been shown that pancreatic beta cells have reduced monocarboxylate transporter 1 (MCT1) expression^44^, likely as a strategy to reduce insulin secretion in response to the elevation of blood lactate levels, especially during exercise. Interestingly, MCT1 was found to be expressed in INS1 cells^45^, which explains their responsiveness to pyruvate stimulation. Beta cells from pancreatic slices, however, had 2-3-fold reduced peak amplitude and approximately ten times reduced frequency of peaks (Fig. 1g, h), likely stemming from a very low level of MCT1 expression in primary beta cells. Despite such a small response to pyruvate stimulation, there was no difference between control and PS1 KD cells in pancreatic slices, strengthening our claim that PS1 plays a role in beta cell glycolysis.

Our previous work showed that mitochondrial respiration and basal mitochondrial ATP were lower in PS1 KD beta cells^18^, but the experiments in those studies were done only with glucose as the main fuel. Current experiments demonstrate that mitochondrial machinery, mitochondrial ability to produce ATP, and downstream ATP-dependent Ca^2+^ signaling are intact in beta cells with reduced PS1 expression. We were able to demonstrate that glycolysis was affected by PS1 KD in all three beta cell models using pyruvate biosensors and measuring stimulated and steady state glycolytic fluxes (Fig. 2). As deficits in glucose uptake could also explain some of these results, we performed a control experiment with glucose biosensor in INS1 cells and observed that PS1 KD doesn’t hamper glucose uptake, but rather increased the glucose accumulation following stimulation, which makes sense in light of reduced glycolytic flux and glycolytic bottleneck at the middle of glycolysis (Supplementary Fig. 1g, h, Fig. 3c).

As we know from our previous work, PS1 controls ER Ca^2+^ leak directed towards mitochondria, and since we concluded that mitochondrial function is not affected when ATP production is directly stimulated by pyruvate, we wondered if hampered glycolysis could be the result of reduced activity of the malate-aspartate shuttle. The reasoning behind this assumption was that if the IMS Ca^2+^ level is also affected by PS1 KD, it could reduce the malate-aspartate shuttle activity, hampering cytosolic NAD^+^/NADH recycling, which in turn would reduce GAPDH activity, as it needs NAD^+^ (Fig. 5g). We tested this assumption by measuring IMS Ca^2+^ level and cytosolic NAD^+^/NADH ratios, and found that PS1 reduces both (Fig. 3 a, Supplementary Fig. 3a). Furthermore, direct testing of steady-state glycolytic metabolites further supported our hypothesis (Fig. 3b, c), as hampered GAPDH activity would result in a glycolytic bottleneck^36^. As a final test of our hypothesis, we asked if we could rescue the glycolytic flux by either overexpressing a component of the malate-aspartate shuttle that could be active at lower Ca^2+^ concentrations or mutating it to make it Ca^2+^ insensitive. Of the two approaches, mutating citrin (AGC2), rescued glycolytic flux and glucose-induced Ca^2+^ oscillations in PS1 KD INS1 cells (Fig. 3d–i). These results strongly support our hypothesis that PS1 works by regulating MAS activity, thereby controlling glycolytic flux of pancreatic beta cells.

Metabolism is a key factor in cell fate determination^46,47^. As we have uncovered a dramatic metabolic phenotype as a result of reduced PS1 expression, we hypothesized that it might have an impact on beta cell identity. Mature beta-cells are identified by the expression of key transcription factors, such as *PDX1, MafA*, and *NKX6.1*, along with the expression of insulin^10,11,48^. On the other hand, *Neurogenin 3* is reported to be expressed in progenitor cells during development, and its expression is decreased upon maturation of beta-cells^10,11,48^. PS1 KD in all three beta-cell models resulted in a different pattern of expression of these key markers, with an overall tendency suggesting a dedifferentiation and a loss of mature beta-cell identity (Fig. 3d–g). The general trend in all three models was a reduction in the expression of insulin, which is regulated by various transcription factors, including *PDX1* and *MafA*. An increase in *Neurogenin 3* expression in PS1 KD INS-1 cells can suggest a step towards progenitor cells, while a decrease in *Neurogenin 3* in EndoC-bH5 and primary mouse pancreatic slices can indicate a further step back in developmental lineage. Such differences might stem from the maturity of cell lines, as EndoC-bH5 cells, per se, were derived from fetal pancreas^22^.

Glycolysis is a centerpiece of cellular glucose metabolism, especially for a cell type that needs to respond to glucose fluctuations. In pancreatic beta cells, accumulation of glycolytic intermediates between fructose-bisphosphate and glyceraldehyde-triphosphate as a result of hyperglycemia was shown to initiate beta cell dedifferentiation by over-activating mTORC1^36^. Our work shows that the same glycolytic intermediates can accumulate as a result of a bottleneck at the GAPDH-catalyzed step due to PS1 KD, despite a reduction in the total glycolytic flux. Thus, we attempted to metabolically reverse the effect of PS1 KD on glycolytic flux, either by supplying extra pyruvate, which rescued Ca^2+^ signaling and ATP production (Fig. 1, Supplementary Fig. 1), or by reducing glucose concentration, which should reduce the accumulation of glycolytic intermediates. From the two interventions, lowering glucose concentration rescued the identity loss in PS1 KD cells (Fig. 4f, g). Furthermore, GAPDH inhibition mimicked PS1 KD and resulted in a similar pattern of identity loss in INS1 cells (Fig. 4e). These results suggest that PS1 contributes to the maintenance of beta cell identity via controlling their glycolytic flux, which we have shown is achieved by regulation of malate-aspartate shuttle activity (Fig. 3). Thus, current work emphasizes a complex role of glycolysis in beta cell metabolism and identity, and places PS1 as a regulator of both.

Several recently published works have linked elevated glycolytic flux and increased mTORC1 activation as a cause for beta cell dysfunction and identity loss^36,37,49^. Our work doesn’t provide a direct link between PS1 and mTORC1 activity. Although we observed an increase in mTORC1 activation in PS1 KD INS1 cells (Supplementary Fig. 4e), application of rapamycin only partially rescued the loss of identity (Supplementary Fig. 4f, g), making it difficult to claim mTORC1 as a downstream effector in identity loss caused by PS1 downregulation. Further studies are warranted to investigate the interrelation of PS1 and mTORC1 in pancreatic beta cells, especially since mTORC1 hyperactivity is also implicated in dementia and AD^50,51^.

It has been reported that diabetes is a risk factor for dementia, including Alzheimer’s disease^13,16,42,43^. In fact, the two diseases share very common metabolic signatures^13^. Given that PS1 mutations are the major cause of familial AD^52^, we investigated the consequences of PS1 mutations in the pancreatic beta cells, as it could give us a better understanding of the protein’s role in both organs and diseases. The triple transgenic mouse model of AD used in this study contains human PS1 M146V mutant knock-in protein^40,41^, reported to cause loss of ER Ca^2+^ leak function^17^, thus we suspected that it would mimic some of the PS1 KD phenotypes we have observed. In line with our experiments with transient KD of PS1 (Supplementary Fig. 1c–e), islets from 3xTg FAD model mice resulted in delayed and reduced glucose-induced Ca^2+^ oscillations (Fig. 1a–c). A delay in the onset of Ca^2+^ oscillations can have substantial implications, such as delayed initial insulin release (Fig. 1d). Although we did not observe significant changes in prolonged insulin release, insulin content, and blood glucose concentration in the 3xTg AD model, delayed or reduced initial phase of insulin release (Fig. 1d–f) could point to an onset of diabetes^53,54^. Indeed, previous research has demonstrated progressive impairment of glucose homeostasis and changes in fasting insulin in the 3xTg AD model at timepoints coinciding with the age used in the current study^55,56^. As our study offers mechanistic insights into the islet autonomous mechanism of metabolic control by PS1, further studies are warranted to put these findings into the context of AD pathology and whole-body metabolism in the AD mouse model. We speculate that beta cell-specific effects of PS1 mutation add a layer of complexity in understanding the impact of AD driver mutations on whole-body metabolism and should be taken in consideration along with possible adaptive mechanisms and comorbidities.

Interestingly, one report also detected the presence of human APP/amyloid beta in the pancreas of the 3xTg AD model mice^55^. Whether or not these potential APP/amyloid beta deposits contribute to beta cell metabolic changes in concert with the PS1 mutation requires further research. Finally, since the loss of beta cell identity is a hallmark of diabetes^8,11^, we investigated if there is an indication of an onset of a loss of identity in the islets of AD model mice, similar to beta cells with transient KD of PS1. Expression analysis of beta cell identity genes revealed a trend pointing towards a loss of beta cell identity in the pancreas of 3xTg mice (Supplementary Fig. 5b), in line with previous observations of reduced pancreatic insulin expression^56^. We assumed that the lack of a more prominent loss of identity phenotype in transgenic knock-in models might stem from the development of compensatory mechanisms to preserve beta cell identity, while in vitro models don’t have enough time for that. Further studies are necessary to investigate these aspects in more detail.

In summary, the current study identifies novel roles of PS1 in pancreatic beta cells, which include control of glucose metabolism through regulation of malate-aspartate shuttle activity at the inner mitochondrial membrane. Furthermore, we found that PS1 exerts pressure on beta cell identity preservation via controlling the glycolytic flux. Thus, current work provides a new perspective into the role of PS1, a risk factor of AD, in systemic glucose metabolism, opening potential research and therapeutic avenues.

## methods

### Animals

All protocols were approved by the Federal Ministry of Education, Science and Research of the Republic of Austria (permit number: 2020-0.258.669). C57BL/6 J mice (034830-JAX, Jackson Laboratories) (8-12 weeks old for KD experiments) and 3xTg-AD mice (034830-JAX, Jackson Laboratories) (34–56 weeks old, with same age controls) were used to prepare pancreatic tissue slices. All animals were kept on a 12:12-h light: dark schedule in individually ventilated cages (Allentown LLC).

Breeding pairs of triple transgenic mice (3xTg), carrying the PS1M146V, APPSwe, and tauP301L transgenes (Oddo et al, 2003), were initially purchased from The Jackson Laboratories (JAX), United States of America. Transgenic animals used in this study were bred in the animal facility of the Institute of Molecular Biosciences, Graz, Austria, using the breeding pairs from Charles River Laboratories. The control strain used for the AD disease model was B6129SF2/J, also bred in-house with breeding pairs from Charles River Laboratories. Animals were housed under conditions essentially as described^57^ and all animal experiments were performed in accordance with national and European ethical regulations (Directive 2010/63/EU) and approved by the responsible institutional or government agencies (Bundesministerium fur Wissenschaft, Forschung und Wirtschaft, BMWFW, Austria: BMWFW-66.007/0032-V/3b/2019, GZ 2023-0.483.703 and GZ 2023-0.691.200).

### Cell culture and transfection

The INS-1 832/13 (INS-1) cells were a generous gift from Prof. Dr. Claes B. Wollheim and Dr. Françoise Assimacopoulos-Jeannet (University Medical Center, Geneva, Switzerland). INS-1 cells were cultured in RPMI 1640 containing 10 mM glucose (PubChem CID: 5793) supplemented with 10 mM HEPES (PubChem CID: 23831), 10% fetal calf serum (FCS), 1 mM sodium pyruvate (PubChem CID: 23662274), 50 μM β-mercaptoethanol (PubChem CID: 1567), 1% (v/v) Pen Strep® (ThermoFischer, Vienna, Austria; 10.000 U/L), and 1.25 μg/ml Amphotericin B (ThermoFischer, Vienna, Austria; 250 μg/mL). Cells were used between passage numbers 53 and 68. Human EndoC-bH5 cells were purchased from Human Cell Design, Inc., and cultured according to the manufacturer’s guidelines in culture media with 5.5. mM glucose. All cells were kept in a humidified incubator (37 °C, 5% CO_2_, 95% air).

For all microscopic experiments, INS-1 cells were plated on 30 mm glass coverslips in 6-well plates and transfected at 50–60% confluency with biosensor DNA constructs (1 μg/well) alone or with siRNA against PS1/scramble control (Table 1) by using 3 μl TransFast transfection reagent (Promega, Madison, WI, USA) in 1 mL of serum and antibiotic-free medium for 12–14 h. After that, transfection media was replaced with 2 mL of full culture medium. All experiments were performed 40–45 h after transfection. Human EndoC-bH5 cells were transduced with adenovirus carrying specific PS1 shRNA (Table 1) or scrambled shRNA (VectorBuilder) and cultured for 48 h. Custom adenovirus constructs were generated that carry a reporter (GFP or mCherry) or a genetically encoded biosensor PyronicSF along with scrambled shRNA or shRNA against PS1 along. These adenoviruses were used for EndoC-bH5 and primary mouse pancreatic slices.

**Table 1:** Sequences of siRNA, shRNA, and primers used

siRNA and shRNA sequences		Ref.
siRNA against human, mouse, and rat PS1	UGG UGU GGU UGG UGA AUA UdTdT	
shRNAs against human, mouse, and rat PS1	GACGAAGA GCTGACATTG AAACTCGAGT TTCAATGTCA GCTCTTCGTC, GCTG TGATTTCAGT ATATGATCTC GAGATCATAT ACTGAAATCA CAGC, CCTGTGCCTT ACATTACTCC TCTCGAGAGG AGTAATGTAA GGCACAGG	
**Primers**		
**Rat:**		
PS1	forward: GTACCTCCCTGAATGGACCG, reverse: CGACCAGCATACGAAGTGGA	
Insulin 2	forward: CCCAGGCTTTTGTCAAACAG reverse: GTGCCAAGGTCTGAAGGTC	^10^
PDX1	forward: TTCATCTCCCTTTCCCGTGG reverse: AGTGTAGGCTGTACGGGTCC	
MafA	forward: TCTTTCTGTGAGCGCGG reverse: TCAGAGTCCGAACCGAGG	^10^
NKX6.1	forward: GCACGCTTGGCCTATTCTCT reverse: CGTTCTCCGAAGTCCCCTTG	
NeuroG3	forward: TCCAGACGCAATTTACTCCAG reverse: CTAGTTCTCCGGGCTCAAAAG	^10^
HPRT	Qiagen QuantiTect Primer set	
**Mouse:**		
PS1	forward: GCCATACTGATCGGCCTGTG reverse: CACGGGTCAAAGCTAGCAGA	
Beta-actin	forward: CCAGCCTTCCTTCTTGGGTAT reverse: GGGTGTAAAACGCAGCTCAG	
*Ins2*	forward: CTGCTGGCCCTGCTCTT reverse: AGGTCTGAAGGTCACCTGCT	
*Pdx1*	forward: AAATCCACCAAAGCTCACGC reverse: GGTCAAGTTCAACATCACTGCC	
*MafA*	forward: CTTCCGGGGTCAGAGCTTCGC reverse: GCCGCTTCTGTTTCAGTCGGATG	
*Nkx6-1*	forward: GTCGGACGCCCATCTTCTG reverse: CAGAGAACGTGGGTCTGGTG	
*NeuroG3*		
**Human**		
PS1	forward: GCAGTATCCTCGCTGGTGAAGA reverse: CAGGCTATGGTTGTGTTCCAGTC	
Insulin 1	forward: AGGCCATCAAGCAGATCACT TTCCCCGCACACTAGGTAGA	
PDX1	forward: AAAGCTCACGCGTGGAAAG GGCCGTGAGATGTACTTGTTG	
MafA	forward: GCTTCAGCAAGGAGGAGGTCAT TCTGGAGTTGGCACTTCTCGCT	
NKX6.1	forward: TGGCCTATTCGTTGGGGATG TGTCTCCGAGTCCTGCTTCT	
NeuroG3	forward: TGCGCCGGTAGAAAGGATG GGGCAGGTCACTTCGTCTTC	
HPRT	Qiagen QuantiTect Primer set	

### Live cell imaging of INS-1 and EndoC-bH5 cells

All live-cell microscopy experiments were performed on an Olympus IX73 inverted microscope unless otherwise mentioned. The microscope is equipped with an UApoN340 40× oil immersion objective (Olympus, Japan) and a CCD Retiga R1 camera (Q-imaging, Canada). For illumination, a LedHUB® (Omnicron, Germany) equipped with 340, 385, 455, 470, and 550 nm LEDs in combination with CFP/YFP/RFP (CFP/YFP/mCherry-3X, Semrock, USA) or GFP (GFP-3035D, Semrock, USA) filter set was used. During the measurements cells were continuously perfused by a gravity-based perfusion system (NGFI, Graz, Austria). Data acquisition and control of the fluorescence microscope was performed using Visiview 4.2.01 (Visitron, Germany). Background-subtracted regions of interest were used for analysis. During live cell imaging experiments, cells were continuously perfused with physiological buffer containing 2 mM CaCl, 135 mM NaCl, 1 mM MgCl_2_, 5 mM KCl, 10 mM HEPES, and indicated glucose concentrations with pH adjusted to 7.4 at room temperature.

Basal IMS Ca^2+^ levels were measured and quantified as previously reported by us^5^ using IMS targeted GEM-GECO^26^ biosensor. Absolute Ca^2+^ concentrations were quantified by obtaining minimum and maximum ratio changes of the biosensor in the presence of ionomycin with 0 and 2 mM Ca^2+^, respectively (0 Ca^2+^ buffer contained 100 µM EGTA).

Cytosolic Ca^2+^ oscillations in response to high glucose and pyruvate were measured using FURA-2 AM Ca^2+^ sensitive dye as previously reported^19^. Shortly, FURA2-AM loading was done in a storage buffer (2 mM CaCl, 138 mM NaCl, 1 mM MgCl_2_, 5 mM KCl, 10 mM HEPES, 2.6 mM NaHCO3, 0.44 mM KH_2_PO_4_, amino acid, and vitamin mix, 3 mM glucose, 2 mM L-glutamine, 1% Penicillin/Streptomycin, 1% Fungizone, pH adjusted to 7.4) with 3 µM FURA2-AM for 30 min at room temperature and washed with fresh storage buffer. FURA2-AM was sequentially excited with 340 nm and 385 nm LEDs, and emission was collected with GFP emission filter set.

Mitochondrial ATP was measured using a genetically encoded matrix-targeted AT1.03^58^ biosensor as previously described by us^5^. The sensor was excited with 455 nm LED and emission was collected at 480 nm and 530 nm using a CFP/YFP/RFP filter set and 505dcxr beam-splitter.

Cytosolic glucose was measured using FLII12Pglu-700μδ672 (gift from Wolf Frommer, Addgene plasmid # 17866)^59^. Cells expressing FLII12Pglu-700μδ6 were excited with 455 nm LED and emission collected at 480 nm and 530 nm using a CFP/YFP/RFP filter set and 505dcxr beam-splitter.

Basal pyruvate and pyruvate production in response to 16 mM glucose in INS-1 cells was quantified using genetically encoded pyruvate biosensor Pyronic^20^. Pyruvate production in EndoC-bH5 cells, pyruvate flux in INS-1 cells, and pyruvate flux in primary mouse pancreatic slices was quantified using PyronicSF biosensor^21^. Adenoviral delivery of biosensors and shRNA was used for human EndoC-bH5 cells and primary mouse pancreatic slices. Cells expressing Pyronic were excited with 455 nm LED and emission collected at 480 nm and 530 nm using a CFP/YFP/RFP filter set and 505dcxr beam-splitter. PyronicSF was excited with 470 nm LED and emission collected with a GFP filter set.

Cytosolic NAD^+^/NADH ratio was quantified in INS-1 cells at given glucose concentrations using NAD^+^/NADH ratio biosensor Peredox^28^ as previously described^5^. INS-1 cells expressing Peredox were excited with 385 nm and 550 nm LEDs, and emission was collected using a CFP/YFP/RFP filter set and 565 LPXR beam-splitter (Semrock, USA).

mTORC1 activity was measured using genetically encoded mTORC1 activity biosensor TORCAR^60^. The sensor was excited with 455 nm LED and emission was collected at 480 nm and 530 nm using a CFP/YFP/RFP filter set and 505dcxr beam-splitter. Cells were imaged in the storage buffer.

### Pancreatic tissue slice preparation

Following CO_2_ euthanasia, the mouse abdominal cavity was assessed via laparotomy. 1.9% low melting-point agarose (Lonza, USA) dissolved in extracellular solution (ECS, (in mM): 125 28 NaCl, NaHCO_3_, 6 glucose, 6 lactic acid, 3 myo-inositol, 2.5 KCl, 2 CaCl_2_, 1.25 NaH_2_PO_4_, 1 MgCl_2_, 0.25 ascorbic acid) was infused at 40°C into the pancreas via the distally clamped common bile duct. The pancreas was then cooled, excised, and sliced using a vibratome (VT1000S, Leica Biosystems). The sliced pancreas (140 µm) was maintained in HEPES-ECS (in mM: 125 NaCl, 10 NaHCO_3_, 10 HEPES, 6 glucose, 6 lactic acid, 3 myo-inositol, 2.5 KCl, 2 Na-pyruvate, 2 CaCl_2_, 1.25 NaH_2_PO_4_, 1 MgCl_2_, 0.25 ascorbic acid; titrated to pH 7.4 with 1 M NaOH). All chemicals were obtained from Sigma-Aldrich if not specified.

### Generation of PSEN1 knockdown in pancreatic tissue slices

Pancreatic tissue slices were cultured in RPMI medium without glucose (11879020, Thermo Fisher Scientific) and supplemented with 10% FBS (F7524, Sigma-Aldrich), 1% Penicillin/Streptomycin (15140122, Gibco, 1:100) and 6 mM glucose (G8270, Sigma-Aldrich) for 24–48 h at 37 °C.

To generate a PS1 knockdown in pancreatic tissue slices, we transduced the slices with 5×10^6^ PFU/ml adenovirus carrying specific mouse PS1 shRNA (Ad-GFP-U6-m-PSEN1-shRNA, shADV-269593, Vector Biolabs) or scrambled shRNA (Ad-U6-RNAi-GFP,1122 N, Vector Biolabs) and cultured for 24-48 h.

### Dynamic [Ca^2+^]c imaging in mouse pancreatic tissue slices

Pancreatic tissue slices were loaded with fluorescent Ca^2+^ indicator in HEPES-ECS (6.35 µM Calbryte 520 or 590-AM (AAT Bioquest), 0.03% Pluronic F-127 (w/v), and 36 0.12% dimethylsulfoxide (v/v)).

Dynamic [Ca^2+^]_c_ imaging was performed on a Nikon inverted confocal system (20x, NA 0.95). Each tissue slice was transferred into the imaging chamber that was continuously perfused with warm (34°C) HEPES-buffered extracellular solution supplemented with various glucose and pyruvate concentrations. The acquisition frequency was 30 Hz at 256×256 pixels resolution and approximately 1 µm^2^ pixel size. Fluorescence was excited with a 488 nm laser and detected at 500–700 nm for the Calbryte 520 indicator, or excited with a 561 nm laser and detected at 570–620 nm for the Calbryte 590 indicator, using a GaAsP PMT detector (Nikon A1R). Each islet [Ca^2+^]c dynamic recording lasted ~3000 s (~50 min).

Islets were identified based on the morphology of the tissue and the characteristic glucose response. The analysis was performed as described in Postic et al., in brief, the regions of interest (ROIs) were semi-automatically determined by employing custom-made Python scripts (Python Software Foundation, Wilmington, Delaware, USA). From the selected ROIs, we gather information about the [Ca^2+^]_c_ changes as the light intensity, the spatial coordinates of the ROIs and neighboring ROIs, statistics about the time recording, recording frequency, and pixel size.

Therefore, all the significant changes of [Ca^2+^]_c_ (with z-score>3) at all realistic time scales (limited by frequency and the time of the recording) within each ROI were automatically distilled and annotated as events. The latency to the first glucose-induced event was measured and compared across conditions. Event frequency was quantified as the number of events per minute per ROI.

### Dynamic pyruvate imaging in mouse pancreatic tissue slices

Pancreatic tissue slices were transduced with adenovirus containing scrambled or PS1 shRNA (described above) along with pyronicSF, an intensiometric pyruvate biosensor. The slices were imaged after 24 h on a Leica TCS inverted confocal system (20x, NA 0.75). As before, each tissue slice was transferred into the imaging chamber that was continuously perfused with warm (34°C) HEPES-buffered extracellular solution without lactate and pyruvate supplemented with glucose 6-8 mM and pyruvate transfer inhibitors UK 5099 and AR-C155858 (4186 and 4960, Tocris). The acquisition frequency was 0.2 Hz at 256×256 pixels resolution and approximately 1 µm2 pixel size. PyronicSF was excited by 490 nm line of a white laser, and the emitted light was detected by the Leica HyD hybrid detector in the range of 500–700 nm using a photon counting mode (Leica Microsystems, Germany).

The recordings of the pyruvate sensor in pancreatic organ slices were analyzed by ImageJ. First, we corrected the recording for the bleaching. Islet mask was defined based on the high-resolution image, where islet borders are clear. The created mask was overimposed on the time course recording of the pyruvate indicator and used to define the ROIs. The mean intensity was measured from the selected ROIs and used for further analysis.

### Quantitative PCR in INS-1 and EndoC-bH5 cells

Total mRNA was isolated using RNeasy® Mini Kit (Qiagen, Hilden, Germany), and reverse transcription was done using Applied Biosystems High-Capacity cDNA Reverse Transcription kit (Thermo Fisher Scientific Baltics UAB, Vilnus, Lithuania). qPCR was performed using Promega GOTaq® qPCR Master Mix (Madison, WI, USA). Primers used for quantification of expression of specific genes are listed in Table 1. HPRT1 was used for normalization. Primers were designed using the NCBI primer design tool unless otherwise referenced otherwise.

### Quantitative PCR in primary pancreatic islets

RNA was transcribed into cDNA using Luna script RT supermix (NEB) according to the Manufacturer’s instructions. For qPCR analysis, 4 µl cDNA were mixed with 1 µl of respective forward and reverse primers, 6 µl Fresenius H_2_O, and 8 µl SYBR green (Bio-Rad) Target gene expression was calculated by the ΔΔCT method. β-actin was used as a housekeeping gene.

### Western blot

For WB analysis, cells were seeded on 10 cm dishes, transfected with respective siRNAs, and harvested 48 h post-transfection. Primary antibodies were used at 1:1000 dilution: rabbit mAb Presenilin1 (D39D1, CST), mouse mAb b-Actin (sc-81178). Secondary antibodies were used at a 1:5000 dilution: goat-anti-rabbit (sc-2054), m-IgGk BP-HRP (sc-516102). Broad Range (10–250 kDa) Color Prestained Protein Standard ladder (NEB, P7719S) was used in all blots.

### RT-PCR from the mouse pancreatic tissue slice

Mouse pancreatic tissue slices were examined under a stereomicroscope, allowing for morphological assessment of the slices and identification of islets. Pancreatic slices containing the islets were used in the experiments. Following the incubation, we isolated the total RNA from pancreatic tissue slices (20 slices per condition) with Total RNA Kit, peqGOLD according to the manufacturer’s instructions (13-6834-02 P, VWR). RNA was measured and adjusted to 500 ng/ml, which was utilized in the reverse transcription reaction (4000 units, 10338842, INV) alongside dNTPs (100 mM each, R0181, Thermo Fisher) and random hexamers (50 µM, N8080127, Invitrogen). RT-PCR was performed for PS1 (fw: GCCATACTGATCGGCCTGTG; rv: CACGGGTCAAAGCTAGCAGA) using KAPA SYBR(R) FAST (KK4600, SIG) on the CFX Connect^TM^Real-Time System (Bio-Rad) and the following cycling parameters 20 s 95 °C, 20 sec 55 °C and 1 s 72 °C for 40 cycles.

Samples were corrected for the total RNA input by normalizing CT values to the CT value of the β-actin (fw: CCAGCCTTCCTTCTTGGGTAT; rv: GGGTGTAAAACGCAGCTCAG).

### Immunofluorescence imaging

For blocking, the pancreatic tissue slices were incubated with blocking buffer (PBS containing 5% BSA (A2153, Sigma Aldrich)) supplemented with 0.1% Triton X-100 (37240, Serva Electrophoresis GmbH) for 1 hour at room temperature. After removal of the blocking solution, primary antibody was diluted in blocking buffer and added in the following concentrations: guinea-pig anti-insulin (ab7842, abcam, 1:100), rabbit anti-glucagon (A14609, ABclonal, 1:100), mouse anti-neurogenin (sc-374442, Santa Cruz Biotechnology 1:200), rabbit anti-PDX1 (5679S, Cell Signaling, 1:200), rabbit anti-MAFA (79737S, Cell Signaling, 1:500), rabbit anti-NKX6.1 (Cell Signaling, 54551S, 1:400). The slides were incubated with the primary antibody overnight at 4 °C. The Secondary antibody incubation lasted for 1 h at RT. Secondary antibodies were diluted in blocking buffer as follows: goat anti guinea-pig Alexa Flour 488 (A-11073, Invitrogen, 1:500), goat anti-rabbit Alexa Flour 647 (A-21244, Invitrogen, 1:500), goat anti-mouse DyLight 550 (SA510173, Sigma-Aldrich, 1:500). To label the nuclei, slices were incubated with 0.1 µg/ml DAPI (6335.1, Carl Roth). Images were obtained on a confocal microscope (Nikon A1R), using the excitation lasers at 405 nm, 488 nm, 561 nm and 640 nm and the GaAsP PMT detectors at 425–475 nm (DAPI), 500-550 nm (Alexa 488) 570–620 nm (DyLight 550) and 663-738 nm (Alexa 647). The possible crosstalk between the channels was minimized by sequential scanning. The slices were imaged with the z-stack mode, reaching about 30 µm in the tissue.

Each of the acquired optical planes was segmented into the ROIs representing single cells based on the DAPI staining with a custom-made ImageJ macro. The macro was developed in ImageJ for more information visit github.com/BHochreiter.

For each physical slice in a z-stack, we then choose the most representative optical plane: for each channel, we construct an average cumulative density function (CDF) of the log ROI intensities, as the median across optical planes. We then calculate the Kolmogorov-Smirnov (KS) distance of each of the optical planes, for each of the channels, as the maximal difference of the CDF from the average one. We sum the KS distances over channels and minimize over optical planes to identify the one that has the CDF closest to the average CDF in all the channels. In most cases, this happens to be the middle optical plane.

For each channel, we then attempt to separate the background and signal ROIs: First, we infer the background intensity by fitting the distribution of log ROI intensities to a two-component Gaussian, and choosing the lower mean value as the central background value to which we normalize all the values. This reduces to a simple subtraction in the log-intensity space. Finally, we use the fitted mixture of Gaussians to binarize the ROIs into background and signal, based on their intensity.

### Mass spectrometry

INS-1 cells were harvested and lysed in a 40:40:20 (V:V:V) mix of acetonitrile:methanol:water, extract was directly used for measurement. LC-MS Data was acquired on a Shimadzu LCMS-8060 system employing a ZICpHILIC (Merck) column (2.1 ×150 mm, 5 µm particle size). The flow rate was kept constant at 200 µL/min, Solvent A was H_2_O, 20 mM NH_4_(H)CO_3_, pH = 9.4, solvent B was acetonitrile, 20 mM NH_4_(H)CO_3_, pH = 9.4. The following gradient was run: 0 min: 90% B; 14 min: 60% B; 20 min: 95% B; 23 min: 95% B; 23.01 min: 90% B, equilibration for 7 min, total run time 30 min. The triple quadrupole was operated in MRM mode, observing the following transitions: 179.0 → 89.1 and 179.0 → 59.1 for glucose; 259.0 → 96.9 and 259.0 → 78.9 for glucose-6-p; 338.9 → 97.0 and 338.9. → 79.0 for fructosebisphosphate; 169.3 → 79.0 and 169.3 → 97.0 for glyceraldehyde-3-phosphate; 89.4 → 43.1 and 89.4 → 41.0 for lactate. Analysis was performed in Skyline 64-bit (vers. 21.2.0.369).

### Isolation of pancreatic islets

For isolation of pancreatic islets, 3 ml of collagenase P (1.7 U/mg; Roche) resolved in HBSS buffer (Gibco, Thermo Fisher Scientific™) supplemented with 4.1 mmol NaHCO_3_ (Merck), was injected into the common bile duct. The pancreas was collected and incubated for 12-16 minutes (depending on the potency of the collagenase) at 37 °C. The collagenase activity was stopped using HBSS (Gibco, Thermo Fisher Scientific™) containing 10% FCS and 4.1 mmol NaHCO_3_. After several washing steps using HBSS (Gibco, Thermo Fisher Scientific™) supplemented with 4.1 mmol NaHCO_3_, a density gradient centrifugation was performed using Histopaque®-1077 (Sigma-Aldrich). The enriched islets were then purified by several washing steps using HBSS. Pancreatic islets were handpicked under a microscope and used for gene expression analysis or glucose-stimulated insulin secretion.

### Glucose-stimulated insulin secretion and insulin content in isolated pancreatic islets

Pancreatic islets were cultured in RPMI media supplemented with 10% FCS (Capricorn Scientific) and 1% penicillin/streptomycin mix (penicillin 10,000 U and 10 mg streptomycin/ml) (Sigma-Aldrich) overnight. Ten pancreatic islets were pre-incubated in Krebs-Henseleit Buffer (KHB), 0.5% BSA, and 2.8 mM glucose for 60 min at 37°C. Thereafter, islets were incubated in KHB containing 0.5% BSA, and 2.8 mM or 16.7 mM glucose for 5 min (initial insulin secretion) or for 30 min at 37 °C. Total insulin content was measured following acid/ethanol (0.18 mM HCl in 70% ethanol) extraction from isolated islets. Insulin levels were determined using the Ultra-Sensitive Mouse Insulin ELISA Kit (Crystal Chem).

### Data analysis

The number of independent experiments is indicated in each figure legend along with the used statistical test and *p*-value. For single-cell experiments, individual cells were used for analysis. Statistical analyses, including Student’s t-test and Analysis of variance (ANOVA) with Tukey post hoc test, were performed on GraphPad Prism software version 9.5.1 (GraphPad Software, San Diego, CA, USA) and Microsoft Excel (Microsoft Office 2013).

### Reporting summary

Further information on research design is available in the Nature Portfolio Reporting Summary linked to this article.

## Supplementary information

Transparent Peer Review file

Supplementary information

Description of Additional Supplementary Files

Supplementary Data

Reporting Summary

## Data Availability

The data supporting the findings of this research can be obtained from the authors upon a reasonable request; please refer to the author’s contributions for details regarding specific datasets. Numerical source data underlying graphs in this article can be found in Supplementary data file. Supplementary Figs. and figure legends are included in the Supplementary file.

## Code availability

The code used in this study is available from the authors on reasonable request.
