# Supplementary material for: Presenilin-1 controls glycolysis and identity of pancreatic beta cells
Source: Commun Biol. 2026 Jul 1;9:1315. doi: 10.1038/s42003-026-10581-6 (PMC13649773; doi:10.1038/s42003-026-10581-6)
Supplement: Supplementary file 1 [file 42003_2026_10581_MOESM1_ESM.pdf]

nature portfolio

Peer Review File

Presenilin-1 controls glycolysis and identity of pancreatic beta cells

Corresponding Author: Professor Wolfgang Graier

This file contains all editorial decision letters in order by version, followed by all author rebuttals in order by version.

Version 0:

Decision Letter:

** Please ensure you delete the link to your author homepage in this email if you wish to forward it to your coauthors **

Dear Professor Graier,

Your manuscript entitled "Presenilin-1 controls glycolysis and identity of pancreatic beta cells" has now been seen by 3 referees, whose comments are appended below. You will see from their comments copied below that while they find your work of potential interest, they have raised quite substantial concerns that must be addressed. In light of these comments, we cannot accept the manuscript for publication, but would be interested in considering a revised version that addresses these concerns.

We hope you will find the referees' comments useful as you decide how to proceed. Should further experimental data or analysis allow you to address these criticisms, we would be happy to look at a substantially revised manuscript. In particular, please note that addressing the following comments will be necessary before we can contact the referees again:
- Addressing concerns regarding the global mutation and potential systemic effects in the AD mouse model, and to provide further characterization of its metabolic phenotype.
- Directly assessing metabolic flux and beta cell function.
- Addressing concerns regarding inconsistent responses and differences in maturity across the models.

We are committed to providing a fair and constructive peer-review process. Do not hesitate to contact us if you wish to discuss the revision or if there are specific requests from the reviewers that you believe are technically impossible or unlikely to yield a meaningful outcome.

If you decide to submit a revised version, we ask that your manuscript complies with our editorial policies. Specifically:

For all graphs depicting a single point value (e.g., mean) with error bars, you must add individual data points or convert the graph to a boxplot or dot-plot to show data distribution.

It’s mandatory to provide access to the numerical source data for graphs and charts either through a repository or by providing the data in a Supplementary Data file (in excel format).

All blots/gels must be accompanied by size markers in every figure panel. Uncropped and unedited blot/gel images must be included as Supplementary Figure(s) in the Supplementary Information pdf.

Please ensure that you have complied with the data deposition policies at the Nature Portfolio, please see here.

Please ensure that you have complied with our policies on research involving animals and humans, see here

Please follow the ARRIVE guidelines for reporting animal experiments. Please fully complete an ARRIVE checklist including both the essential and recommended set of items (adding information to the manuscript where needed) and upload this with

your revised manuscript.

Please also see our revision checklist for guidance on formatting the manuscript and complying with our policies. A comprehensive guide to our formatting requirements for final submissions is also available for your reference here.

Please use the following link to submit your revised manuscript, point-by-point response to the referees' comments (which should be in a separate document to any cover letter) and any completed checklist:

Link Redacted

This url links to your confidential home page and associated information about manuscripts you may have submitted or be reviewing for us. If you wish to forward this email to co-authors, please delete the link to your homepage first

We hope to receive your revised manuscript within 3 months, but appreciate that every situation is unique. Please take as long as necessary to address these concerns in full, including performing any additional experimental work required. We look forward to receiving your revised manuscript when it is ready and will not enforce any specific deadline. However, please bear in mind that if the revision process takes significantly longer than six months, we may take into account whether anything similar has been accepted for publication at Communications Biology or published elsewhere in the meantime.

Please do not hesitate to contact me if you have any questions or would like to discuss the required revisions further. Thank you for the opportunity to review your work.

Best regards,

Soona Shin, PhD
Editorial Board Member
Communications Biology
orcid.org/0000-0001-5696-9505

Reviewers' comments:

Reviewer #2 (Remarks to the Author):

In the submitted manuscript the authors explored the link between AD and impaired beta-cell function, focusing on a role for presenilin-1 (PS1). In the study they used a combination of in vivo and in vitro models including cells of human and rodent origin. Many of the techniques are state of the art and all are performed well with appropriate statistical power. Using these models, they show that PS1 controls glycolytic flux by maintaining sub mitochondrial Ca^{2+} homeostasis, with PS1 knockdown causing a bottleneck at the GAPDH step. They show that this bottleneck disrupts β cell identity which could be partially reversed by inhibiting mTORC1 activity. Islets/slices isolated from an Alzheimer's disease mouse model show some similarities to the in vitro studies, although the model retains glucose homeostasis. This is a nicely performed studies that addresses a key question and will be a key contribution to the research field. Several aspects should be addressed to improve the quality of the manuscript:

1) The authors report that pyruvate had similar effects on Ca2+ oscillations in control and PS1 KD cells. The authors only show the traces for the INS model but not for the mouse slice model. Is this because different results were obtained? The results in figure 1d vs figure 1f seem to differ with respect to their response to pyruvate. The mouse slice data shown in figure 1f seems to suggest a much lower response to pyruvate vs glucose, unlike the INS1 model. This is not surprising given that pyruvate enters the cells via MCT transporters and it is well established that beta-cells have limited MCT1 expression (DOI: 10.4161/isl.2.2.11025), primarily to prevent insulin release in response to excess lactate production, e.g. during exercise. The authors do show that the INS1 model respond to pyruvate, which may be explained by some beta-cell lines showing altered ability for pyruvate uptake vs primary tissue (doi: 10.3390/cells11152330). The authors should include all data for the mouse slice studies and should describe and discuss any differences between the models. Perhaps the authors could use their pyruvate biosensors to confirm uptake of pyruvate into the INS1 model vs the mouse slices, if relevant?

2) The authors utilise a 3-way inhibitory tactic to assess steady state glycolytic flux (Figure 2c+g). Did the authors confirm that these inhibitors did indeed inhibit pyruvate utilisation in the INS1 model? It is unlikely that the LDH inhibitor would have a dramatic effect given the low LDH activity noted in INS1 cells (PMID: 8106462) although the authors were able to measure lactate using mass spec so perhaps their cells behave slightly differently. It is also not clear why only the glucose data is shown for the EndoC model (figure 2e) and only the inhibitor data for the mouse slice model (figure 2g). What is the reasoning behind this? It is quite confusing to determine which models are used for each panel as this is not clearly stated in the text and at times feels misleading. In addition, how are the values in the bar charts calculated? They sometimes resemble the F/Fo changes in the traces (i.e. figure 2a+b) but sometimes do not (figures 2g+h). This authors should address these issues.

3) The authors should include images for the slice cultures for all of the proteins measured (as in figure 4c) within the supplementary data.

4) The differences in neurogenin3 expression in the INS1 vs EndoC models may represent a difference in maturity of the cell lines. The EndoC cells were derived from fetal pancreas and thus may demonstrate a less mature phenotype. The authors should consider this possibility and refer to it in the paper if they deem it relevant.

5) Timepoints for all conditions should be included in the legends, particularly for the changes in glucose concentration (Figure 4).

6) The authors note that the effect of rapamycin is partial. This is true for the INS1 model in figure 4g. The effect of rapamycin looks more than partial in the EndoC model. Could the authors speculate on why this may be? Was there any difference in the culture of these cells during these experiments or does it reflect an inherent difference between the cell lines?

7) The authors conclude that the 3Tg AD model shows normal glucose tolerance despite a deficiency in Ca2+ signalling and early stage GSIS. However, glucose tolerance was only assessed using a random blood glucose measurement which does not provide an accurate estimation of glucose tolerance. Also, given that the mutations were global, it is possible that changes in insulin sensitivity could compensate for changes in insulin release. Importantly, the authors do not state the age of the mice. This should be noted in the figure legends and in the method section. This is particularly relevant given their discussion around the potential progression towards diabetes in the 3Tg AD model. Where does the age of the mice in this study fit with the referenced papers noting diabetes progression?

8) For the analysis of calcium imaging for the mouse slices, how did the authors identify the pancreatic islets away from the acinar tissue? Was this based on glucose responsiveness? Handpicking of the ROIs was mentioned for the pyruvate imaging. How was this established? The authors should provide additional detail here to inform understanding of the data presented.

9) In the methods, this sentence is unclear: Mouse pancreatic tissue slices were examined and the ones containing were incubated as described.

Reviewer #3 (Remarks to the Author):

Major findings: The authors identifies a novel role of PS1 in pancreatic beta cells, demonstrating that PS1 loss impairs glycolytic flux upstream of mitochondria metabolism, leading to defective glucose induce Ca signaling and partial loss of beta cell identity. The authors propose that PS1 regulates ER-mito Ca coupling, thereby maintaining MAS activity and cytosolic NAD regeneration required for sustained glycolysis. The study combines multiple beta cell models and extends the findings to AD mouse model, suggesting a potential link between AD associated PS1 mutations and beta cell dysfunction.
Questions:
1. PS1 loss is known to impair ER Ca leak and may lead to ER Ca overload, which can trigger ER stress or alter Ca signaling that might suppress glycolysis. In the current study, ER Ca level and ER stress markers are not assessed. How do the authors distinguish the proposed IMS Ca/MAS mediated mechanism from alternative consequences of ER Ca overload following PS1 KD
2. Throughout the study, glucose induced Ca oscillation are used as a primary functional readout or beta cell performance, whereas direct measurements of GSIS are limited. While Ca signaling is an important upstream event, is does not always predict insulin secretion. Why did the authors rely on glucose induce Ca oscillation rather than GSIS as the main functional outcome to assess beta cell dysfunction?
3. The involvement of mTORC1 in beta cell identity loss if briefly addressed and supported by limited evidence, with rapamycin showing only partial rescue of marker expression and no functional readouts. Can the authors provide stronger biochemical or functional evidence linking mRORC1 activation to beta cell dysfunction downstream of PS1 loss, or should this aspect be present more cautiously?
4. The AD mouse model carries whole body PS1 mutation, introducing potential systemic and developmental confounders when interpreting beta cell intrinsic mechanism. Moreover, defects appear largely restricted to early Ca responses and first phase insulin secretion, while prolong GSIS and blood glucose are mostly preserved. How do the authors justify the use of a systemic PS1 mutation to support a beta cell autonomous mechanism and how should the modest in vivo phenotype be interpreted in terms of physiological relevance?

Reviewer #4 (Remarks to the Author):

This study identifies a role of PS1 in the control of beta cell glucose metabolism through regulation of malate-aspartate shuttle activity at the inner mitochondrial membrane. The authors conclude that PS1 exerts pressure on beta cell identity preservation via controlling the glycolytic flux. This conclusion is based on the following results: (1) PS1 is important for glucose metabolism preceding mitochondrial entry of pyruvate in pancreatic beta cells; (2) PS1 controls glycolytic flux in pancreatic beta cells; (3) PS1 controls the glycolytic flux by regulating malate-aspartate shuttle activity; (4) PS1 preserves beta cell identity by maintaining glycolytic flux; (5) biomarker dynamics in the early onset familial Alzheimer’s Disease (FAD) cause impaired glucose responsiveness in pancreatic islets.

The results are novel and of interest to the community and across disciplines, including metabolism and neurodegeneration. Further evidence is required to strengthen the conclusions. A summary of my main comments is provided below:
1. Knockdown of PS1 impaired glucose-induced cytosolic Ca^{2+} oscillations in INS-1 cells and pancreatic organ slices, as demonstrated by a delayed onset and reduced frequency of Ca^{2+} spikes following glucose stimulation (Fig. 1). The authors conclude that PS1 is required for efficient glucose sensing upstream of mitochondrial pyruvate oxidation, but is dispensable once pyruvate directly fuels mitochondrial metabolism. This conclusion is weakened by the indirect Ca^{2+} measurements rather than direct metabolic flux analysis. While the glucose-versus-pyruvate comparison is suggestive, it does not definitively localize the metabolic defect.
2. Knockdown of PS1 reduced glucose-induced increases in cytosolic pyruvate in INS-1 cells, as measured using the Pyronic sensor, indicating impaired glycolytic output (Fig. 2). These findings were replicated in human EndoC-βH5 cells and primary pancreatic slices, where PS1 depletion attenuated both glucose-stimulated cytosolic pyruvate dynamics and calculated glycolytic flux. The conclusion is based on cytosolic pyruvate accumulation as an indirect proxy. While Pyronic sensors report cytosolic pyruvate dynamics, these measurements cannot distinguish whether reduced pyruvate reflects: impaired glycolysis, increased pyruvate consumption or mitochondrial uptake, or altered pyruvate transport across the plasma or mitochondrial membranes. Additionally, the use of PS1 knockdown may have off-target or compensatory effects on β-cell metabolism. Direct measurements of glycolytic flux (glucose tracing) would be needed to definitively establish that PS1 controls glycolysis per se rather than indirectly affecting cytosolic pyruvate levels.
3. Knockdown of PS1 in INS-1 cells disrupted glucose-stimulated cytosolic NAD^+/NADH dynamics, consistent with impaired malate-aspartate shuttle (MAS) activity (Fig. 3). Mass spectrometry analysis of glycolytic intermediates revealed accumulation upstream of the MAS, suggesting a glycolytic bottleneck in PS1-deficient cells. It is proposed that PS1 maintains glycolytic flux by regulating MAS activity, linking cytosolic redox balance to efficient glucose metabolism and β-cell Ca^{2+} signaling. Similarly, the conclusion relies on indirect readouts. Direct flux measurements and functional validation of MAS transporter activity are required to establish that PS1 regulates glycolytic flux via the MAS.
4. Knockdown of PS1 in INS-1 cells and primary pancreatic slices led to a marked reduction in in insulin, PDX1, MafA, and NKX6.1, along with an increase in the progenitor marker Neurogenin-3 (Fig. 4). It is concluded that PS1 is essential for maintaining β-cell differentiation and functional identity, linking metabolic regulation to transcriptional programs that preserve the mature β-cell state. While reductions in insulin, PDX1, MafA, and NKX6.1 suggest dedifferentiation, there is no direct assessment of β-cell functionality.
5. Pancreatic islets from the 3xTg familial Alzheimer's disease (FAD) mice exhibited impaired glucose responsiveness compared to wild-type controls (Fig. 5). Cytosolic Ca^{2+} oscillations in response to 8 mM glucose were delayed in onset and reduced in peak frequency, indicating blunted β-cell excitability. First-phase insulin secretion during the initial 5 minutes of glucose stimulation was significantly decreased, while total insulin content remained unchanged, and extended 30-minute secretion was moderately affected. These data suggest that PS1 mutations (a genetic driver of FAD) compromise β-cell glucose sensing and early insulin secretory responses, consistent with a role for PS1-mediated regulation of β-cell metabolism upstream of mitochondrial pyruvate utilization. However, the link between FAD genetic mutations and impaired β-cell glucose responsiveness is largely correlative. Developmental changes in islet composition, altered paracrine signaling, or systemic metabolic differences in the 3xTg mice, could contribute to the observed phenotype. Functional measurements focus on ex vivo islets, without in vivo validation of glucose tolerance. Targeted PS1 modulation, or assessment of glycolytic and mitochondrial flux, may help validate this potential brain-pancreas link in FAD.

** See the Nature Portfolio author and referees' website at www.nature.com/authors for information about policies, services and author benefits

Communications Biology is committed to improving transparency in authorship. As part of our efforts in this direction, we are now requesting that all authors identified as 'corresponding author' create and link their Open Researcher and Contributor Identifier (ORCID) with their account on the Manuscript Tracking System prior to acceptance. ORCID helps the scientific community achieve unambiguous attribution of all scholarly contributions. You can create and link your ORCID from the home page of the Manuscript Tracking System by clicking on 'Modify my Springer Nature account' and following the instructions in the link below. Please also inform all co-authors that they can add their ORCIDs to their accounts and that they must do so prior to acceptance.
https://www.springernature.com/gp/researchers/orcid/orcid-for-nature-research

For more information please visit http://www.springernature.com/orcid

If you experience problems in linking your ORCID, please contact the Platform Support Helpdesk.

Version 1:

Decision Letter:

** Please ensure you delete the link to your author homepage in this email if you wish to forward it to your coauthors **

Dear Professor Graier,

Your manuscript entitled "Presenilin-1 controls glycolysis and identity of pancreatic beta cells" has now been seen again by our referees, whose comments appear below. In light of their advice I am delighted to say that we are happy, in principle, to publish a suitably revised version in Communications Biology.

We therefore invite you to revise your paper one last time to address the remaining concerns of our reviewers. At the same time we ask that you edit your manuscript to comply with our format requirements and to maximise the accessibility and therefore the impact of your work.

* Please see the attached document for editorial requests for the final version (.docx file). Please ensure a completed version of this file is uploaded as a Related Manuscript File with your final submission.

* Please review our final submission file checklist to ensure all necessary files are present with your final submission and to avoid delays in accepting your manuscript. For your reference, a style and formatting guide is available here and includes all of our style requirements.

It is important that you pay careful attention to the requests in these documents to avoid a delay in formal acceptance of the article.

If you have any questions or concerns about any of our requests, please do not hesitate to contact me.

Open access
Communications Biology is a fully open access journal. Articles are made freely accessible on publication. For further information about article processing charges, open access funding, and advice and support from Nature Research, please visit https://www.nature.com/commsbio/open-access
At acceptance, you will be provided with instructions for completing the open access licence agreement on behalf of all authors. This grants us the necessary permissions to publish your paper. Additionally, you will be asked to declare that all required third party permissions have been obtained, and to provide billing information in order to pay the article-processing charge (APC).

Please use the following link to upload your revised files:

Link Redacted

** This url links to your confidential home page and associated information about manuscripts you may have submitted or be reviewing for us. If you wish to forward this email to co-authors, please delete the link to your homepage first **

We hope to hear from you within two weeks. If you expect the process to take longer than one month, please let us know.

Congratulations on an excellent paper!

Best regards,

Soona Shin, PhD
Editorial Board Member
Communications Biology
orcid.org/0000-0001-5696-9505

Joao Manuel de Sousa Valente, PhD
Senior Editor
Communications Biology
orcid.org/0000-0003-1888-6324

PS: At acceptance, the corresponding author will be provided with instructions for completing the license on behalf of all authors. This grants us the necessary permissions to publish your paper. Additionally, you will be asked to declare that all required third party permissions have been obtained, and to provide billing information in order to pay the article-processing charge (APC).

REVIEWERS' COMMENTS:

Reviewer #2 (Remarks to the Author):

My concerns have been adequately addressed.

Reviewer #3 (Remarks to the Author):

Thank you for addressing my questions.
- The new data on XBP1 splicing and ER calcium helps clear up the concerns about ER stress.
- While some of the mechanisms (like the mTORC1 pathway and the whole body mouse model results) are still a bit weak or relies on speculation, I understand your reasoning and accept your explanations for this stage of the study.
- I am happy to recommend acceptance of the manuscript, but please make sure the following points are clearly addressed in your final text and framed as areas for further wor
- mTORC1 pathway (Q3): The WB shows a very small change in p-P70S6K. Please make sure the final text clearly states that the role of mTORC1 is not conclusive yet and downstream of mTORC1 is not only p-P70S6K, there is needed to be properly investigated in future studies.
- In vivo phenotype (Q4): The difference between the strong effects in cells and the mild effects in the actual mice needs a clear mention in the Discussion. Please explicitly state that whole body adaptations or other co-morbidities are just hypotheses for now and will need to be tested in future work.

Reviewer #4 (Remarks to the Author):

I would like to thank the authors for their consistent effort to address my questions.
The revisions strengthened both the mechanistic interpretation and experimental support of the study. The authors performed additional complementary experiments, including NMR-based metabolomic analyses and fluorescence biosensor assays, to address concerns regarding the indirect nature of several readouts.
Although direct isotope tracing and transporter-specific MAS flux measurements were not feasible within the revision timeframe, the newly added data consistently support the central conclusion that PS1 regulates β-cell glycolytic flux through modulation of malate-aspartate shuttle activity. Importantly, independent methodologies converged on the same findings, including impaired glucose-to-lactate flux, altered NAD+/NADH balance, accumulation of upstream glycolytic intermediates, reduced aspartate levels, and defective pyruvate dynamics in PS1-deficient cells.
Although the authors clarified the interpretation and limitations of the 3xTg FAD mouse data, emphasizing that the current study was designed primarily to define the β-cell autonomous metabolic role of PS1 rather than whole-body metabolic phenotypes is needed in the Introduction part of the manuscript.
While some aspects, particularly in vivo validation and direct metabolic flux tracing, warrant future investigation, the revised manuscript now provides substantially stronger evidence supporting a mechanistic link between PS1, MAS-dependent glycolytic regulation, and preservation of β-cell identity and function.

** See the Nature Portfolio author and referees' website at www.nature.com/authors for information about policies, services and author benefits

Open Access This Peer Review File is licensed under a Creative Commons Attribution 4.0 International License, which permits use, sharing, adaptation, distribution and reproduction in any medium or format, as long as you give appropriate credit to the original author(s) and the source, provide a link to the Creative Commons license, and indicate if changes were made.
In cases where reviewers are anonymous, credit should be given to 'Anonymous Referee' and the source.
The images or other third party material in this Peer Review File are included in the article's Creative Commons license, unless indicated otherwise in a credit line to the material. If material is not included in the article's Creative Commons license and your intended use is not permitted by statutory regulation or exceeds the permitted use, you will need to obtain permission directly from the copyright holder.
To view a copy of this license, visit https://creativecommons.org/licenses/by/4.0/

To the editor

We are grateful for the opportunity to revise our manuscript with insightful comments from the reviewers. We have addressed all of the comments and suggestions given by the reviewers to the best of our ability, given the time restraints. We believe the changes have enriched the manuscript and made it more focused.

- Addressing concerns regarding the global mutation and potential systemic effects in the AD mouse model, and to provide further characterization of its metabolic phenotype.

Since the experiments from the mouse model were done on isolated islets and organ slices, we believe they serve as a tool to investigate islet/beta-cell autonomous mechanism of the role of PS1 and the impact of AD causing mutations. We have addressed this point by shifting the focus of the manuscript towards mechanistic insight into beta cell metabolism, rather than focusing on whole body metabolism. We believe that further metabolic characterization of the metabolic phenotype of the AD mouse model would require a substantial amount of work and would deserve separate manuscript. Knowing the beta cell autonomous effect of AD causing mutations would help us and others decipher and better attribute various metabolic phenotypes observed in this AD model organism. In fact, we are currently working on the impact of AD causing mutations on another organ system/cell type, which complements the current study and will hopefully give more clarity on some of the aspects observed by us and other scientists using the 3xTg AD mouse model.

- Directly assessing metabolic flux and beta cell function.

We have performed additional metabolic flux analysis using NMR. Furthermore, we have supplemented the manuscript with further flux assays using microscopy-based techniques.

- Addressing concerns regarding inconsistent responses and differences in maturity across the models.

We have addressed the concerns regarding differences in response among models.

Overall, we hope the editor and the reviewers find our efforts satisfactory.

Reviewer #2 (Remarks to the Author):

In the submitted manuscript the authors explored the link between AD and impaired beta-cell function, focusing on a role for presenilin-1 (PS1). In the study they used a combination of in vivo and in vitro models including cells of human and rodent origin. Many of the techniques are state of the art and all are performed well with appropriate statistical power. Using these models, they show that PS1 controls glycolytic flux by maintaining sub mitochondrial Ca^{2+} homeostasis, with PS1 knockdown causing a bottleneck at the GAPDH step. They show that this bottleneck disrupts β cell identity which could be partially reversed by inhibiting mTORC1 activity. Islets/slices isolated from an Alzheimer's disease mouse model show some similarities to the in vitro studies, although the model retains glucose homeostasis. This is a nicely performed studies that addresses a key question and will be a key contribution to the research field. Several aspects should be addressed to improve the quality of the manuscript:

We thank the reviewer for their fair assessment of the manuscript and useful suggestions. Please find below our point-by-point response to the aspects raised.

1) The authors report that pyruvate had similar effects on Ca2+ oscillations in control and PS1 KD cells. The authors only show the traces for the INS model but not for the mouse slice model. Is this because different results were obtained? The results in figure 1d vs figure 1f seem to differ with respect to their response to pyruvate. The mouse slice data shown in figure 1f seems to suggest a much lower response to pyruvate vs glucose, unlike the INS1 model. This is not surprising given that pyruvate enters the cells via MCT transporters and it is well established that beta-cells have limited MCT1 expression (DOI: 10.4161/isl.2.2.11025), primarily to prevent insulin release in response to excess lactate production, e.g. during exercise. The authors do show that the INS1 model respond to pyruvate, which may be explained by some beta-cell lines showing altered ability for pyruvate uptake vs primary tissue (doi: 10.3390/cells11152330). The authors should include all data for the mouse slice studies and should describe and discuss any differences between the models. Perhaps the authors could use their pyruvate biosensors to confirm uptake of pyruvate into the INS1 model vs the mouse slices, if relevant?

Response: We thank the referee for this comment. We now have included representative traces from mouse slice model in figure 1. It can be seen that the response to pyruvate stimulation is different compared to INS1 cells, likely due to lower expression of MCT1 in primary islets, as suggested by the reviewer and shown in the cited publication. Stimulation with 5 mM pyruvate in primary pancreatic slices results in smaller Ca2+ peaks compared to glucose stimulation, with no significant differences observed between control and PS1 KD slices. A short section in discussion part was added to address the potential differences between models (lines 339-348).

2) The authors utilise a 3-way inhibitory tactic to assess steady state glycolytic flux (Figure 2c+g). Did the authors confirm that these inhibitors did indeed inhibit pyruvate utilisation in the INS1 model? It is unlikely that the LDH inhibitor would have a dramatic effect given the low LDH activity noted in INS1 cells (PMID: 8106462) although the authors were able to measure lactate using mass spec so perhaps their cells behave slightly differently. It is also not clear why only the glucose data is shown for the EndoC model (figure 2e) and only the inhibitor data for the mouse slice model (figure 2g). What is the reasoning behind this? It is quite confusing to determine which models are used for each panel as this is not clearly stated in the text and at times feels misleading. In addition, how are the values in the bar charts calculated? They sometimes resemble the F/Fo changes in the traces (i.e. figure 2a+b) but sometimes do not (figures 2g+h). This authors should address these issues.

Response: We thank the referee for bringing up these aspects. Indeed, there are several publications arguing the expression of LDH in INS1 and primary pancreatic beta cells (PMID: 38607916). As we have observed lactate in MS data, and later on in NMR data, we wanted to make sure to inhibit all the escape routes for pyruvate by inhibiting LDH, MPC and MCT. Both high glucose and inhibitor protocols were applied to INS1 cells, while only high glucose was used on EndoC model, as this is a commercial human beta cell line, only select experiments were performed on these cells to confirm key experiments. On the primary slices, increasing glucose only (within physiological range that was enough to see calcium oscillations) didn't result in signal change that was detectable using the biosensor and the optical set up. To overcome this, we went for a protocol that, in our opinion, would have yielded the maximal pyruvate accumulation, i.e., increasing glucose concentration and blocking the pyruvate exit routes.

Furthermore, as per the reviewer's comment, we have labeled the panels with the respective models used (Figure 2). F/F_0 has been used when utilizing the intensiometric pyruvate sensor (panels 2 c-h), while R/R_0 was used for panel 2 a. The reason for normalizing the ratio signal was for clarity, as the signal changes are not big, and we haven't observed changes in basal pyruvate levels (Supplementary figure 2a).

3) The authors should include images for the slice cultures for all of the proteins measured (as in figure 4c) within the supplementary data.

Response: As suggested by the referee**,** representative images of slice cultures corresponding to figure 4 c have been added to supplementary figure 4.

4) The differences in neurogenin3 expression in the INS1 vs EndoC models may represent a difference in the maturity of the cell lines. The EndoC cells were derived

from fetal pancreas and thus may demonstrate a less mature phenotype. The authors should consider this possibility and refer to it in the paper if they deem it relevant.

Response: We thank the referee for this insightful and important comment. We agree with the reviewer that EndoC-bH5 cell line shows an opposite change in Ngn3 expression upon PS1 KD. The expression levels of Ngn3 or any other gene cannot be compared between cell lines, but only within each cell line. As the reviewer suggests, the opposite manner of Ngn3 change could stem from the maturity of cell lines used. As per the reviewer's comment, the difference in neurogenin 3 expression, as well as the different pattern of change in expression of various identity genes, has been discussed in the discussion section (lines 374-386):

5) Timepoints for all conditions should be included in the legends, particularly for the changes in glucose concentration (Figure 4).
Response: As suggested by the reviewer, the figure legends for figure 4 and supplementary figure 4 (as we have moved experiments with rapamycin to supplementary figures) have been adjusted to clarify the duration/timeframe of different treatments, including lowering glucose concentration.

6) The authors note that the effect of rapamycin is partial. This is true for the INS1 model in figure 4g. The effect of rapamycin looks more than partial in the EndoC model. Could the authors speculate on why this may be? Was there any difference in the culture of these cells during these experiments or does it reflect an inherent difference between the cell lines?

Response: We agree that rapamycin had a stronger effect in the EndoC model, and even resulted in increased neurogenin 3 expression in PS1 KD cells compared to controls. The increase in Neurogenin 3 expression is not viewed as a positive outcome, hence we reasoned that the rescue wasn't complete even in EndoC-bH5 cells. The main reason behind our statement was based on the fact that lowering culture glucose resulted in a more complete rescue in both cell lines, thus, compared to low glucose, rapamycin had a weaker, partial rescue. As the reviewer noted in comment #4, we suspect that the differential effect of rapamycin might partially be due to intrinsic differences between the cell lines. Furthermore, the mTORC1 biosensor showed that using rapamycin lowers mTORC1 activity in both control and PS1 KD cells, as expected. Although mTORC1 activity is expected to be low in beta cells and increased mTORC1 activity was shown to be associated with loss of identity (PMID: 31265435, PMID: 36376280), some basal activity is required for beta cell development and function (PMID: 19690069).

Additionally, another reviewer asked us to evaluate mTORC1 activity using biochemical approach. Thus, we have performed a Western blot analysis of P70 S6 kinase phosphorylation, a downstream target of mTORC1 activity. WB showed us a very minor effect of PS1 KD on S6 kinase phosphorylation.

Taking into account all of the mentioned points, we thought that the effect of rapamycin on beta cell identity might be misleading and difficult to interpret in correlation with PS1. As a result, we have weakened our point on the involvement of mTORC1 downstream of PS1 and tried to put it as a possible mechanism that requires further investigation (mentioned in lines 258-264, 402-408).

7) The authors conclude that the 3Tg AD model shows normal glucose tolerance despite a deficiency in Ca2+ signalling and early stage GSIS. However, glucose tolerance was only assessed using a random blood glucose measurement which does not provide an accurate estimation of glucose tolerance. Also, given that the mutations were global, it is possible that changes in insulin sensitivity could compensate for changes in insulin release. Importantly, the authors do not state the age of the mice. This should be noted in the figure legends and in the method section. This is particularly relevant given their discussion around the potential progression towards diabetes in the 3Tg AD model. Where does the age of the mice in this study fit with the referenced papers noting diabetes progression?

Response: Thank you for these important comments. The 3xTg mice used in the study were 34-56 weeks old (age added in materials and methods section, lines 500-502). Unfortunately, no real glucose tolerance test was performed, and as the reviewer notes, the blood glucose concentration wasn't significantly altered in ad libitum fed mutant mice. Others have done proper glucose tolerance tests in the same mice strain and found that 3xTg mice get deteriorating glucose tolerance at around 10-14 months of age, and show increased fasted insulin levels at around same age (PMID: 26108977). As the reviewer points out, the mentioned study suggests a defect in insulin sensitivity in the 3xTg model, which might complicate our conclusions on the islet/beta-cells autonomous role of PS1 when applied on the whole body glucose metabolism. We believe that investigating the role of AD causing mutations on the whole-body glucose metabolism would require substantial amount of work. As similar concerns regarding the in vivo model have been raised by other reviewers as well, we would like to mention that we are carrying out additional work that focuses on other organ systems in these transgenic mice, which might make the picture more complete. Thus, the focus of the current work is more mechanistic, and the in vivo model is mainly used to assess beta-cell autonomous phenotype; hence, we mainly focus on isolated islets, and not on whole-body phenotype, which would help future studies in assessing the systemic phenotypes of AD causing mutations. As a result, we tried to shift the focus more

towards beta cell/islet autonomous view throughout the manuscript. We hope for the reviewer's understanding in this regard and are open for further suggestions.

8) For the analysis of calcium imaging for the mouse slices, how did the authors identify the pancreatic islets away from the acinar tissue? Was this based on glucose responsiveness? Handpicking of the ROIs was mentioned for the pyruvate imaging. How was this established? The authors should provide additional detail here to inform understanding of the data presented.

Response: Following clarifications have been added to the relevant section of materials and methods: In the calcium imaging of pancreatic tissue slices islets were identified based on the morphology of the tissue and the characteristic glucose response (line 604).

The recordings of pyruvate sensor in pancreatic organ slices were analyzed by ImageJ. First, we corrected the recording for the bleaching. Islet mask was defined based on the high-resolution image where islet boarders are clear. The created mask was over imposed on the time course recording of the pyruvate indicator and used to define the ROIs. The mean intensity was measured from the selected ROIs and used for further analysis (lines 625-629).

9) In the methods, this sentence is unclear: Mouse pancreatic tissue slices were examined and the ones containing were incubated as described.

Response: Following clarifications have been added to the relevant section of materials and methods (lines 651-653):
Mouse pancreatic tissue slices were examined under stereomicroscope, allowing for morphological assessment of the slices and identification of islets. Pancreatic slices containing the islets were used in the experiments.

Reviewer #3 (Remarks to the Author):

Major findings: The authors identifies a novel role of PS1 in pancreatic beta cells, demonstrating that PS1 loss impairs glycolytic flux upstream of mitochondria metabolism, leading to defective glucose induce Ca signaling and partial loss of beta cell identity. The authors propose that PS1 regulates ER-mito Ca coupling, thereby maintaining MAS activity and cytosolic NAD regeneration required for sustained glycolysis. The study combines multiple beta cell models and extends the findings to AD

mouse model, suggesting a potential link between AD associated PS1 mutations and beta cell dysfunction.

The thank the reviewer for fair assessment of the manuscript and useful suggestions, which we have tried to address below.

Questions:

1. PS1 loss is known to impair ER Ca leak and may lead to ER Ca overload, which can trigger ER stress or alter Ca signaling that might suppress glycolysis. In the current study, ER Ca level and ER stress markers are not assessed. How do the authors distinguish the proposed IMS Ca/MAS mediated mechanism from alternative consequences of ER Ca overload following PS1 KD

Response: As suggested by the reviewer, we have assessed basal ER calcium level and ER stress indicator in control and PS1 KD INS1 cells. We found a slight increase in ER Ca2+ load, in line with reduced calcium leak, but no indication of ER stress, as XBP1 splicing was not altered (new data added to Supplementary Figure 3 b, d). Cells likely respond to reduced ER Ca2+ leak by reducing ER Ca2+ uptake to maintain a Ca2+ homeostasis, and although there is a slight increase in basal Ca2+ load, it doesn't seem to lead to ER stress. Furthermore, we haven't observed changes in basal cytosolic Ca2+ levels in our previous work (PMID: 31529929), and also have quantified it now, and saw no difference (Supplementary figure 3 c), suggesting the PS1 mediated leak is directed at mitochondria. Lines 156-161 added.

2. Throughout the study, glucose induced Ca oscillation are used as a primary functional readout or beta cell performance, whereas direct measurements of GSIS are limited. While Ca signaling is an important upstream event, is does not always predict insulin secretion. Why did the authors rely on glucose induce Ca oscillation rather than GSIS as the main functional outcome to assess beta cell dysfunction?

Response: We agree with the reviewer that glucose induced Ca2+ oscillations are a proxy of the real GSIS measurement, but we have shown insulin secretion in INS1 and Min6 cells with PS1 KD and in primary islets with GSK3b inhibition (essential for PS1 function in beta cells) in our previous work (PMID: 31529929 and some figure panels attached below). Accordingly, in the current work, we have added insulin secretion data from islets isolated from WT and 3xTg mice model (Figure 5). We hope for the reviewer's understanding for our reasoning not to repeat the data we have already published.

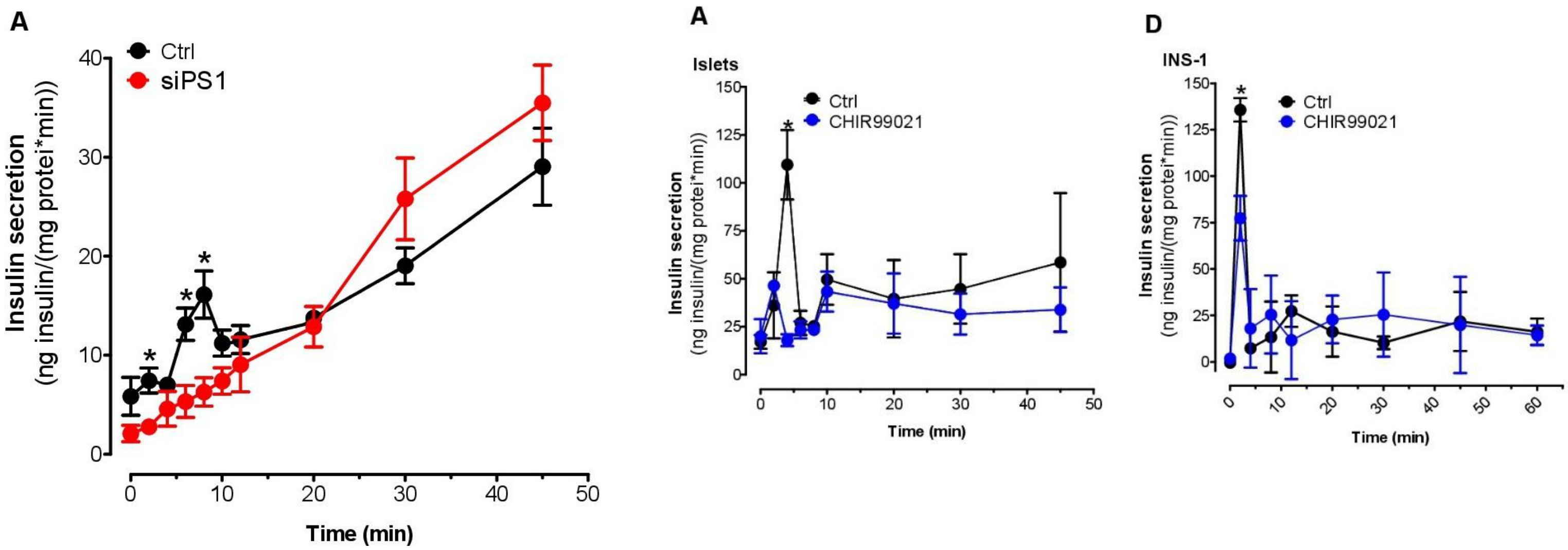

Figure panels from PMID:31529929

3. The involvement of mTORC1 in beta cell identity loss if briefly addressed and supported by limited evidence, with rapamycin showing only partial rescue of marker expression and no functional readouts. Can the authors provide stronger biochemical or functional evidence linking mRORC1 activation to beta cell dysfunction downstream of PS1 loss, or should this aspect be present more cautiously?

Response: We thank the reviewer for this comment. We have attempted to perform additional experiments to evaluate the change in mTORC1 activity in PS1 KD INS1 cells using Western blot to assess P70 S6 kinase phosphorylation, a downstream target of mTORC1 activity. The WB did not show a strong change in the phosphorylation of P70S6K in PS1 KD cells (attached below, not added to the manuscript). Given the small degree of change in our measurements using mTORC1 biosensor (supplementary figure 4 f) and the WB, we have decided to weaken our claims regarding mTORC1 acting downstream of PS1 throughout the manuscript. Although we believe mTORC1 might play a role in the PS1 mediated signaling cascade, its direct involvement in the loss of identity is not conclusive (mentioned in lines 258-264, 402-408). We are currently working on a different manuscript focusing on signaling aspect of PS1 in other cell types, where we will further investigate the involvement of mTORC1 in PS1 dependent signaling cascades.

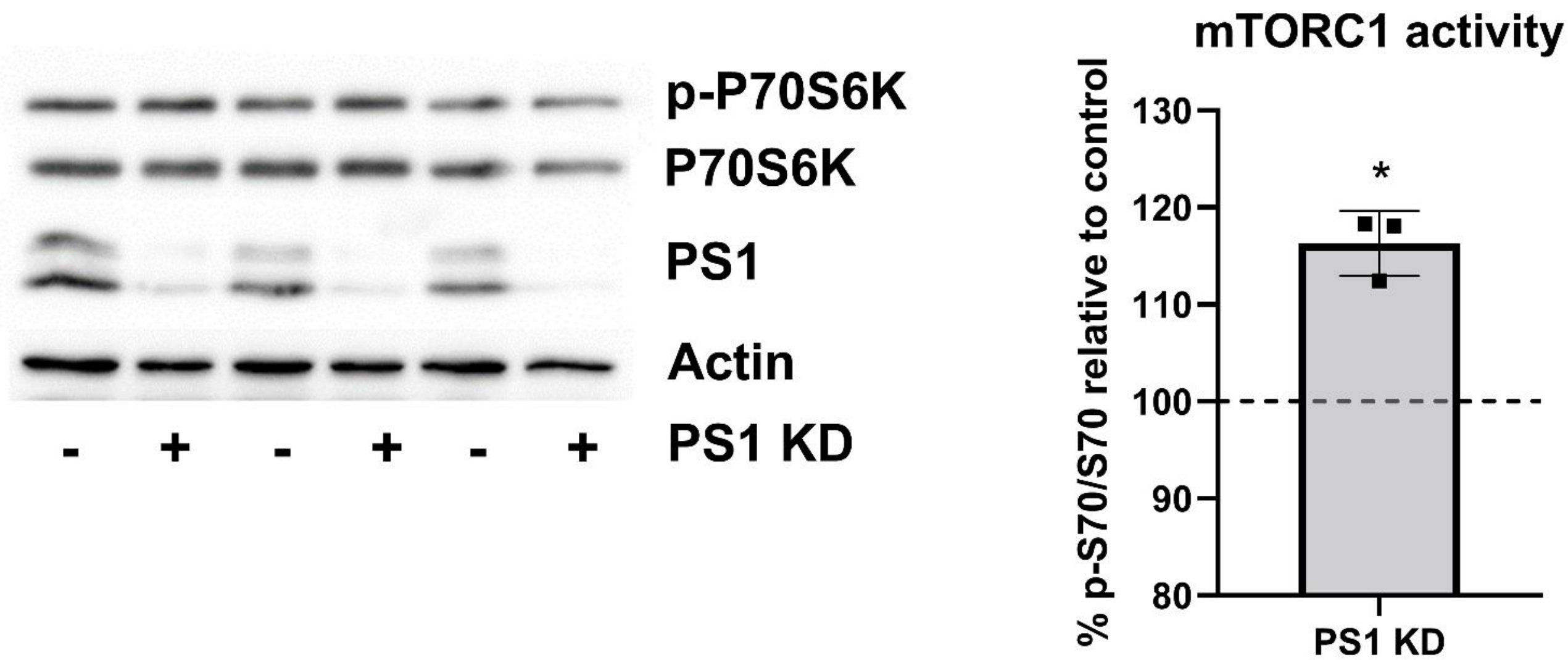

4. The AD mouse model carries whole body PS1 mutation, introducing potential systemic and developmental confounders when interpreting beta cell intrinsic mechanism. Moreover, defects appear largely restricted to early Ca responses and first phase insulin secretion, while prolong GSIS and blood glucose are mostly preserved. How do the authors justify the use of a systemic PS1 mutation to support a beta cell autonomous mechanism and how should the modest in vivo phenotype be interpreted in terms of physiological relevance?

Response: We agree with the reviewer's concern regarding potential systemic effects of PS1 mutation. At the same time, we believe that isolated islets from global PS1 mutant mice show beta cell autonomous phenotypes, in line with beta cell line models and islets/pancreatic slices with transient PS1 KD. As the reviewer correctly notes, the modest in vivo phenotype likely stems from whole body adaptation and possible compensatory mechanisms. We speculate that some co-morbidities can exacerbate this phenotype, and we are currently investigating this possibility in an ongoing study. Overall, we hope that the combination of transient PS1 KD on primary islets and beta cell lines is enough to convince the reviewer that the phenotypes observed in islets from 3xTg mouse model are stemming from beta cell autonomous effect, while we are speculating on the reasons for modest in vivo phenotype and plan future work to address this.

Reviewer #4 (Remarks to the Author):

This study identifies a role of PS1 in the control of beta cell glucose metabolism through regulation of malate-aspartate shuttle activity at the inner mitochondrial membrane. The

authors conclude that PS1 exerts pressure on beta cell identity preservation via controlling the glycolytic flux. This conclusion is based on the following results: (1) PS1 is important for glucose metabolism preceding mitochondrial entry of pyruvate in pancreatic beta cells; (2) PS1 controls glycolytic flux in pancreatic beta cells; (3) PS1 controls the glycolytic flux by regulating malate-aspartate shuttle activity; (4) PS1 preserves beta cell identity by maintaining glycolytic flux; (5) biomarker dynamics in the early onset familial Alzheimer's Disease (FAD) cause impaired glucose responsiveness in pancreatic islets.
The results are novel and of interest to the community and across disciplines, including metabolism and neurodegeneration. Further evidence is required to strengthen the conclusions. A summary of my main comments is provided below:

We thank the reviewer for the fair assessment of the manuscript and their suggestions. We have tried to address the comments raised by the reviewer using the tools at our exposal. As our laboratory is primarily based on fluorescence imaging techniques, we have relied on our collaborators for the flux analysis data. Given the time restraints, we hope the reviewer finds our efforts satisfactory. Where possible, we have attempted to supplement missing flux data with experiments using microscopy. We agree with the reviewer on many points that most of the evidence is indirect, but we have used complementary techniques to achieve such results and believe that when several unrelated methods indirectly give the same result, it is fair to assess these data as strong evidence.

1. Knockdown of PS1 impaired glucose-induced cytosolic Ca^{2+} oscillations in INS-1 cells and pancreatic organ slices, as demonstrated by a delayed onset and reduced frequency of Ca^{2+} spikes following glucose stimulation (Fig. 1). The authors conclude that PS1 is required for efficient glucose sensing upstream of mitochondrial pyruvate oxidation, but is dispensable once pyruvate directly fuels mitochondrial metabolism. This conclusion is weakened by the indirect Ca^{2+} measurements rather than direct metabolic flux analysis. While the glucose-versus-pyruvate comparison is suggestive, it does not definitively localize the metabolic defect.

Response: To address the points raised by the reviewer (comments 1-3), we have attempted to performed additional metabolomics experiments using NMR. NMR was chosen because we were not able to improve our already existing mass spectrometry experiments and could not detect glycolytic intermediates between GAP and lactate with the technique available in our collaborators' laboratory. Hence, it was suggested by our collaborators to attempt to address these comments using glucose stimulation assay coupled with NMR analysis. Furthermore, as our main expertise lies in microscopy, we tried to supplement some of the reviewer's requests using available fluorescence-based biosensors. NMR assay (Supplementary figure 2 b) showed

decreased glucose to lactate flux upon glucose stimulation in PS1 KD INS1 cells, supporting our previous results using pyruvate biosensor. We also saw that LDHa levels were not changed by PS1 KD (Supplementary figure 2 c), suggesting the reduced lactate production is not due to reduced expression of lactate dehydrogenase (lines 124-130).

Furthermore, the flux analysis showed a drop in citrate levels after glucose stimulation in PS1 KD INS1 cells, while control cells maintained relatively stable citrate levels (Supplementary figure 2 d). This is likely explained by citrate consumption in TCA cycle (boosted by Ca2+ oscillations) and inability of PS1 KD cells to supply enough pyruvate to replenish TCA cycle intermediates. This readout supports our findings that mitochondria per se are not affected by the PS1 KD, but the glycolysis is not keeping up and not supplying enough “fuel” for the TCA cycle due to reduced flux and glycolytic bottleneck.

2. Knockdown of PS1 reduced glucose-induced increases in cytosolic pyruvate in INS-1 cells, as measured using the Pyronic sensor, indicating impaired glycolytic output (Fig. 2). These findings were replicated in human EndoC-βH5 cells and primary pancreatic slices, where PS1 depletion attenuated both glucose-stimulated cytosolic pyruvate dynamics and calculated glycolytic flux. The conclusion is based on cytosolic pyruvate accumulation as an indirect proxy. While Pyronic sensors report cytosolic pyruvate dynamics, these measurements cannot distinguish whether reduced pyruvate reflects: impaired glycolysis, increased pyruvate consumption or mitochondrial uptake, or altered pyruvate transport across the plasma or mitochondrial membranes. Additionally, the use of PS1 knockdown may have off-target or compensatory effects on β-cell metabolism. Direct measurements of glycolytic flux (glucose tracing) would be needed to definitively establish that PS1 controls glycolysis per se rather than indirectly affecting cytosolic pyruvate levels.

Response: We agree that glucose stimulated pyruvate accumulation in the cytosol measured with Pyronic biosensor might have several interpretations. Thus, to address the basal glycolytic flux, we have used pharmacological inhibition of pyruvate exit routes (mitochondrial pyruvate uptake, pyruvate extrusion, and pyruvate consumption by LDH) and observed pyruvate accumulation in the presence of constant glucose (Figure 2 c). This accumulation (or basal glycolytic flux) was lower in PS1 KD cells. Furthermore, as suggested by the reviewer and mentioned above, we have attempted to perform glycolytic flux analysis by NMR using low to high glucose transition. Changes in lactate (end product of glycolysis) and citrate (TCA cycle intermediate) were added in Supplementary Figure 2 and discussed in the previous response. Unfortunately, we were not able to perform glucose tracing experiments in the given timeframe.

3. Knockdown of PS1 in INS-1 cells disrupted glucose-stimulated cytosolic NAD^+/NADH dynamics, consistent with impaired malate-aspartate shuttle (MAS) activity (Fig. 3). Mass spectrometry analysis of glycolytic intermediates revealed accumulation upstream of the MAS, suggesting a glycolytic bottleneck in PS1-deficient cells. It is proposed that PS1 maintains glycolytic flux by regulating MAS activity, linking cytosolic redox balance to efficient glucose metabolism and β-cell Ca^{2+} signaling. Similarly, the conclusion relies on indirect readouts. Direct flux measurements and functional validation of MAS transporter activity are required to establish that PS1 regulates glycolytic flux via the MAS.

Response: We were not able to perform a direct assessment of MAS activity, but tried to supplement our findings using different techniques. NMR analysis showed reduced NAD+ levels in PS1 KD INS1 cells (Supplementary figure 3 e), supporting our results using genetically encoded NAD+/NADH biosensor (lines 165-166). Furthermore, using fructose bisphosphate biosensor (Hylight), we saw increased basal fructose bisphosphate level in PS1 KD INS1 cells (Supplementary figure 3 d), supporting our previous findings using MS (lines 176-177). Additionally, NMR showed reduced aspartate level in PS1 KD INS1 cells (Supplementary figure 3 f), which can be the result of hampered MAS activity (PMID: 30122555) (lines 182-184). We agree with the reviewer that these are again indirect validations of PS1 role in MAS modulation, but as these are found using unrelated tools, we believe they provide a strong support for the involvement of PS1 in MAS regulation. Furthermore, in our submitted version, we had included rescue experiments using citrin (AGC2, MAS component) mutants mimicking Ca2+ bound state, which is in our eyes, is a strong argument on the involvement of MAS in PS1 mediated control of glycolysis.

4. Knockdown of PS1 in INS-1 cells and primary pancreatic slices led to a marked reduction in in insulin, PDX1, MafA, and NKX6.1, along with an increase in the progenitor marker Neurogenin-3 (Fig. 4). It is concluded that PS1 is essential for maintaining β-cell differentiation and functional identity, linking metabolic regulation to transcriptional programs that preserve the mature β-cell state. While reductions in insulin, PDX1, MafA, and NKX6.1 suggest dedifferentiation, there is no direct assessment of β-cell functionality.

Response: We agree with the referee, but we have already measured insulin secretion in INS1 cells with PS1 KD and in primary islets with GSK3b inhibition (essential for PS1 function in beta cells) in our previous work (PMID: 31529929 and attached below). As a proxy for beta cell function, in the current work, we have shown glucose induced calcium measurements (Figure 1). Glucose-induced insulin secretion experiments for the 3xTg mouse model were present in the submitted manuscript (Figure 5).

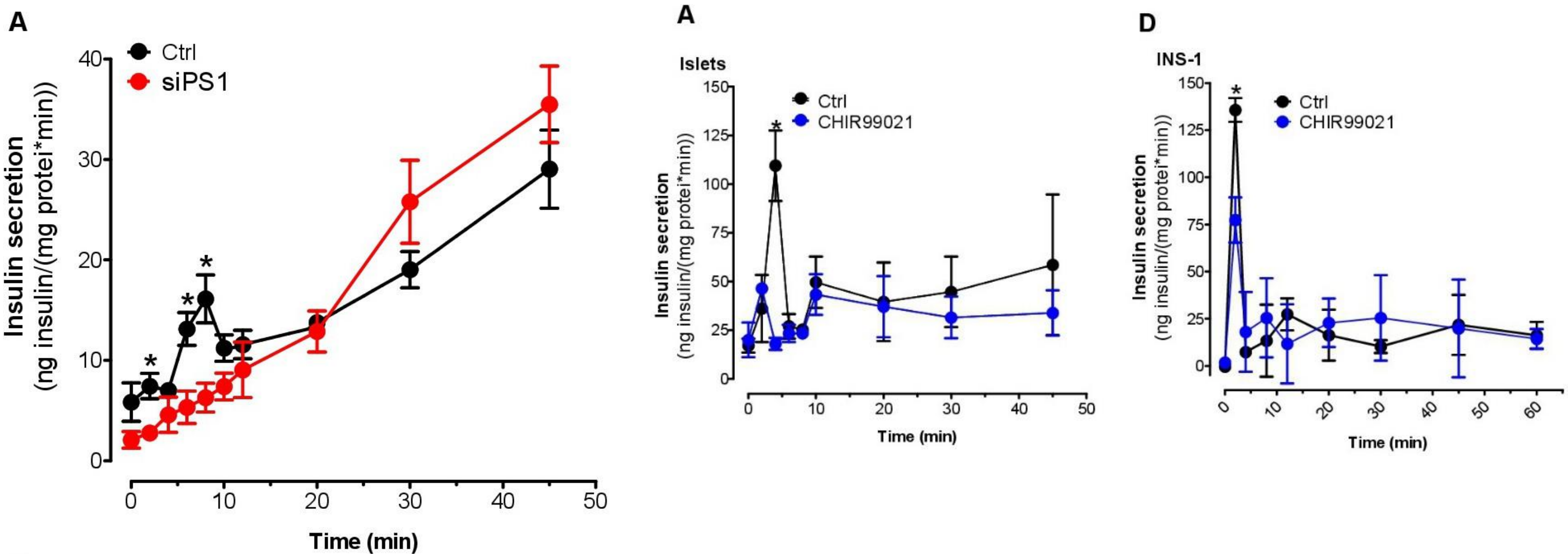

Figure panels from PMID:31529929

5. Pancreatic islets from the 3xTg familial Alzheimer's disease (FAD) mice exhibited impaired glucose responsiveness compared to wild-type controls (Fig. 5). Cytosolic Ca^{2+} oscillations in response to 8 mM glucose were delayed in onset and reduced in peak frequency, indicating blunted β-cell excitability. First-phase insulin secretion during the initial 5 minutes of glucose stimulation was significantly decreased, while total insulin content remained unchanged, and extended 30-minute secretion was moderately affected. These data suggest that PS1 mutations (a genetic driver of FAD) compromise β-cell glucose sensing and early insulin secretory responses, consistent with a role for PS1-mediated regulation of β-cell metabolism upstream of mitochondrial pyruvate utilization. However, the link between FAD genetic mutations and impaired β-cell glucose responsiveness is largely correlative. Developmental changes in islet composition, altered paracrine signaling, or systemic metabolic differences in the 3xTg mice, could contribute to the observed phenotype. Functional measurements focus on ex vivo islets, without in vivo validation of glucose tolerance. Targeted PS1 modulation, or assessment of glycolytic and mitochondrial flux, may help validate this potential brain-pancreas link in FAD.

Response: We agree with the reviewer on the shortcomings of the work in regards to the in vivo model. The main focus of the current work was to focus on mechanistic, beta cell autonomous metabolic role of PS1. We agree that global nature of the PS1 mutation could have had an impact on whole body glucose metabolism. Additionally, the

3xTg mutant strain has been well characterized (PMID: 26108977). Our results add an important piece of mechanistic insight into contribution of PS1 to beta cell metabolism and identity, which should be taken into account when studying the role of PS1 in FAD, as alterations in insulin release and glucose sensitivity of beta cells can have a compounding effect in addition to other age-related diseases. We have done additional experiments as asked by the reviewer in other comments, but think that addressing comment #5 requires a major commitment worthy of a whole new study. Currently, we have ongoing studies focusing on the role of PS1 in other organs/cells, which might add further clarity in the role of PS1 in whole-body metabolism. Thus, at this stage, we are unfortunately unable to fully satisfy the current concern and hope for the reviewer's understanding.

REVIEWERS' COMMENTS:

Reviewer #2 (Remarks to the Author):

My concerns have been adequately addressed.

Reviewer #3 (Remarks to the Author):

Thank you for addressing my questions.
- The new data on XBP1 splicing and ER calcium helps clear up the concerns about ER stress.
- While some of the mechanisms (like the mTORC1 pathway and the whole body mouse model results) are still a bit weak or relies on speculation, I understand your reasoning and accept your explanations for this stage of the study.
- I am happy to recommend acceptance of the manuscript, but please make sure the following points are clearly addressed in your final text and framed as areas for further wor
- mTORC1 pathway (Q3): The WB shows a very small change in p-P70S6K. Please make sure the final text clearly states that the role of mTORC1 is not conclusive yet and downstream of mTORC1 is not only p-P70S6K, there is needed to be properly investigated in future studies.
- In vivo phenotype (Q4): The difference between the strong effects in cells and the mild effects in the actual mice needs a clear mention in the Discussion. Please explicitly state that whole body adaptations or other co-morbidities are just hypotheses for now and will need to be tested in future work.

Response: The suggestions have been addressed (lines 334-341, 356-361)

Reviewer #4 (Remarks to the Author):

I would like to thank the authors for their consistent effort to address my questions.
The revisions strengthened both the mechanistic interpretation and experimental support of the study. The authors performed additional complementary experiments, including NMR-based metabolomic analyses and fluorescence biosensor assays, to address concerns regarding the indirect nature of several readouts.
Although direct isotope tracing and transporter-specific MAS flux measurements were not feasible within the revision timeframe, the newly added data consistently support the central conclusion that PS1 regulates β-cell glycolytic flux through modulation of malate-aspartate shuttle activity. Importantly, independent methodologies converged on the

same findings, including impaired glucose-to-lactate flux, altered NAD+/NADH balance, accumulation of upstream glycolytic intermediates, reduced aspartate levels, and defective pyruvate dynamics in PS1-deficient cells.

Although the authors clarified the interpretation and limitations of the 3xTg FAD mouse data, emphasizing that the current study was designed primarily to define the β-cell autonomous metabolic role of PS1 rather than whole-body metabolic phenotypes is needed in the Introduction part of the manuscript.

While some aspects, particularly in vivo validation and direct metabolic flux tracing, warrant future investigation, the revised manuscript now provides substantially stronger evidence supporting a mechanistic link between PS1, MAS-dependent glycolytic regulation, and preservation of β-cell identity and function.

Response: The suggestions have been addressed (lines 69-70)
