# Supplementary material for: Presenilin-1 controls glycolysis and identity of pancreatic beta cells
Source: Commun Biol. 2026 Jul 1;9:1315. doi: 10.1038/s42003-026-10581-6 (PMC13649773; doi:10.1038/s42003-026-10581-6)
Supplement: Supplementary file 2 [file 42003_2026_10581_MOESM2_ESM.pdf]

SUPPLEMENTAL DATA

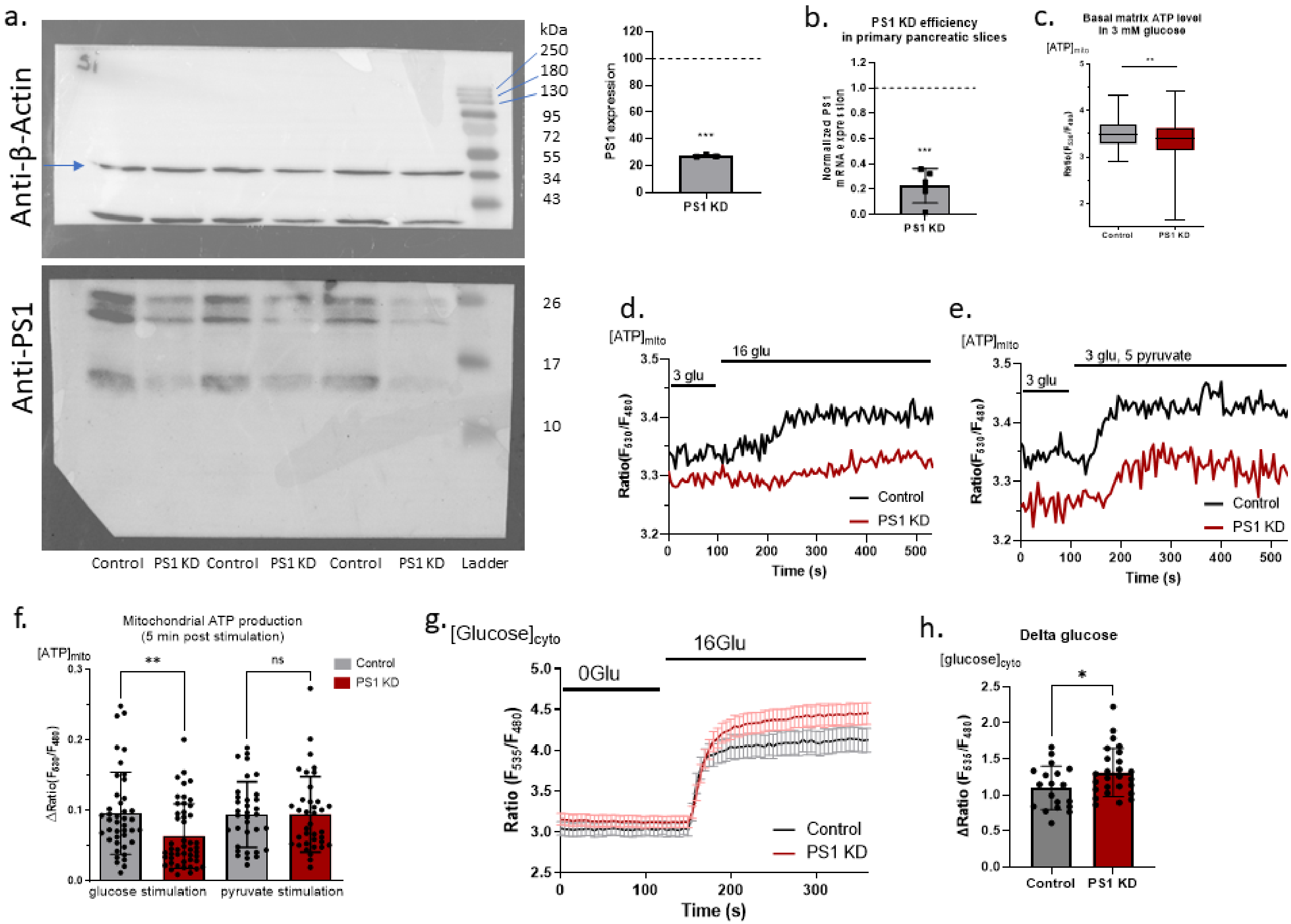

Supplementary figure 1. a, Western blot (upper – cropped, lower - uncropped) and analysis (right) show the PS1 expression in control and PS1 KD INS-1 cells (n=3). **b**, KD efficiency of PS1 in primary mouse pancreatic organ slices, mRNA expression normalized to controls (n=5). **c**, basal mitochondrial matrix ATP level shown as mitochondrial AT1.03[57] emission ratio (+/- SEM) in 3 mM glucose for control and PS1 KD INS-1 cells (control n=85, PS1 KD n=99). **d-e**, representative traces demonstrating mitochondrial ATP dynamics in response to the increase of extracellular glucose from 3 mM to 16 mM (**d**) and the addition of 5 mM pyruvate (**e)** in control and PS1 KD INS-1 cells. **f,** Bar graphs (+/- SEM) show the change in mitochondrial ATP levels after 5 minutes in 16 mM glucose or 5 mM pyruvate, as shown in **d** and **e** (control glucose stim., n=42, PS1 KD glucose stim., n=34, control pyruvate stim., n=48, PS1 KD pyruvate stim., n=37). **g**, cytosolic glucose dynamics (mean +/-SEM) shown as FLII12Pglu-700μδ6 emission ratio upon extracellular glucose addition in control and PS1 KD INS-1 cells. **h**, change in cytosolic glucose level (+/-SD) after addition of 16 mM extracellular glucose as shown in **g** (control, n=19; PS1 KD, n=26). Unpaired t-test (for **c, f, h**), paired t-test (for **a** and **b**), n.s. $p > 0.05$, * $p < 0.05$, ** $p < 0.01$, *** $p < 0.001$

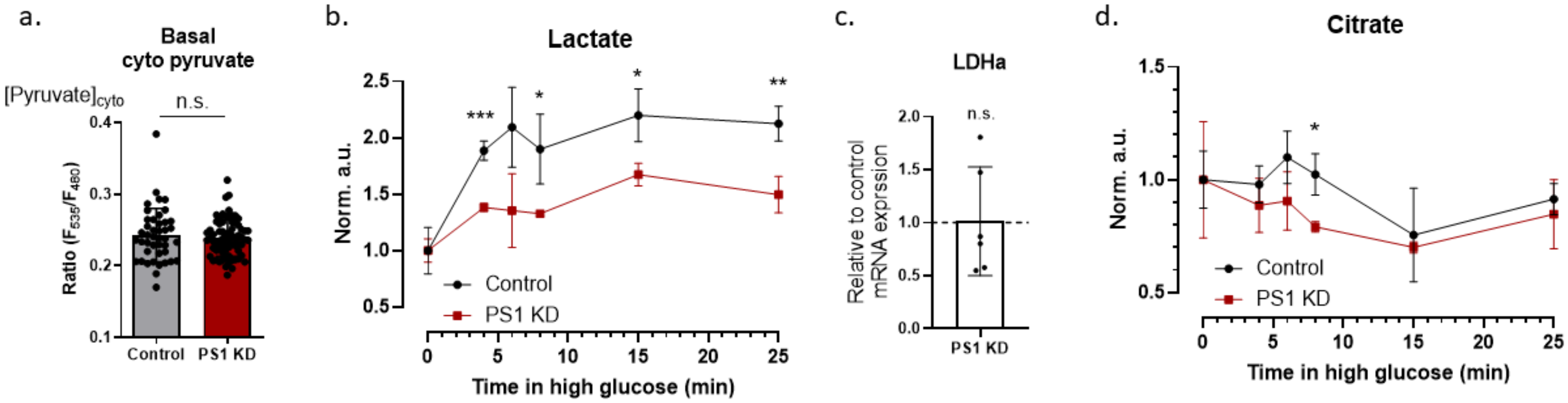

Supplementary figure 2. a, basal cytosolic pyruvate levels shown as Pyronic emission ratio (mean +/-SEM) in control and PS1 KD INS-1 cells (control, n=43; PS1 KD, n=71). **b**, cellular lactate levels (mean ± SD) of INS1 cells represented as normalized NMR intensity units (a.u.) under control (black) and PS1 KD (red) conditions following high glucose (16 mM) exposure (n = 3). **c**, mean ± SD LDHa mRNA expression in PS1 KD INS1 cells normalized to controls (n=6). **d**, cellular citrate levels (mean ± SD) of INS1 cells represented as normalized NMR intensity units (a.u.) under control (black) and PS1 KD (red) conditions following high glucose (16 mM) exposure (n = 3).

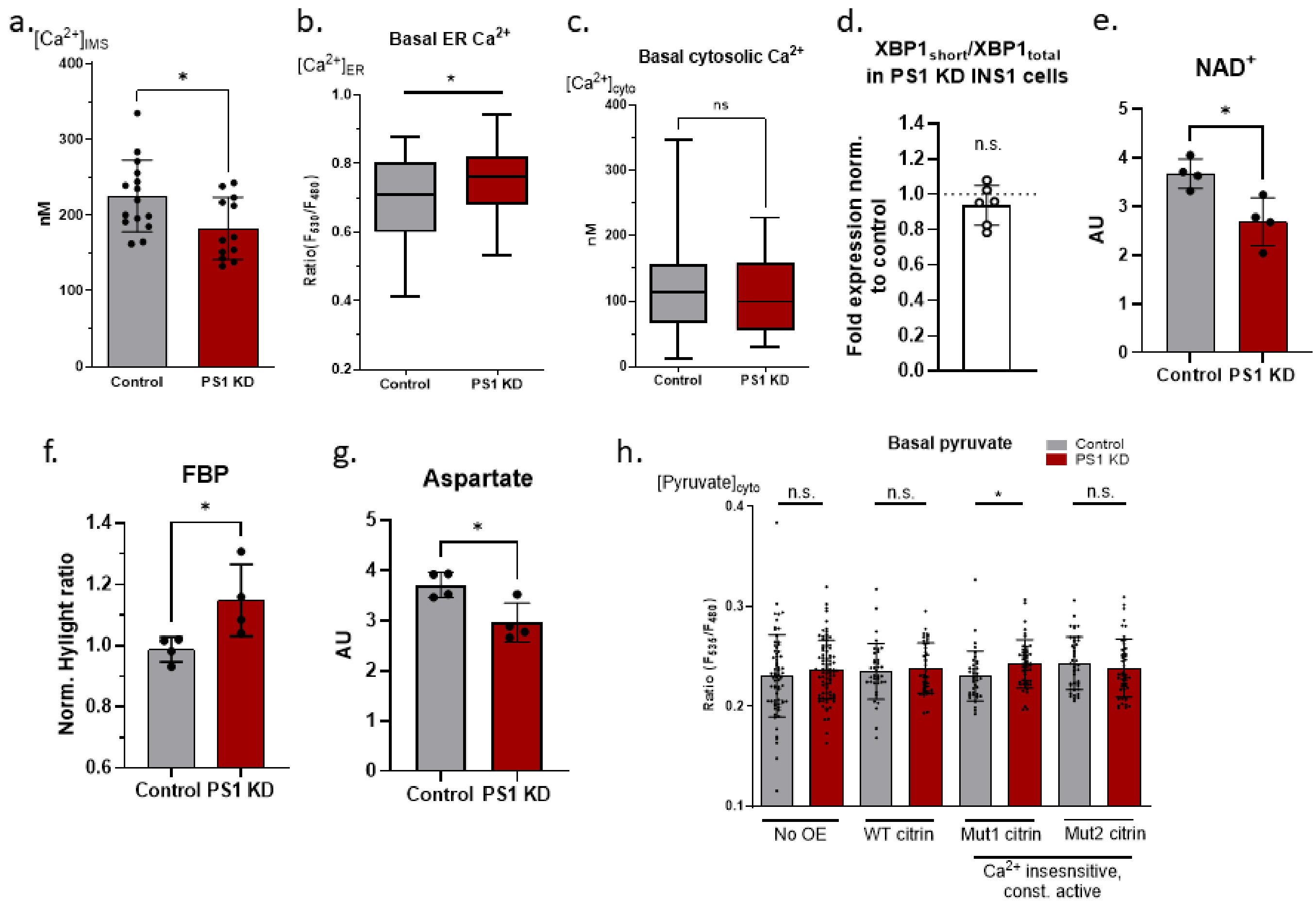

Supplementary figure 3. a-c, graphs (mean +/- SD) depict basal Ca^{2+} concentrations in the mitochondrial IMS (**a**) (control n=15, PS1 KD n=12), ER (**b**) (control n=48, PS1 KD n=53), and cytosol (**c**) (control, n=24, PS1 KD, n=29) of INS-1 cells. **d**, bar graphs show mean ± SD ratio of spliced to total XBP1 transcript levels in PS1 KD INS1 cells normalized to control ratio (n=6). **e**, cellular NAD^+ levels (mean ± SD) of INS1 cells represented as normalized NMR intensity units (a.u.) under control and PS1 KD conditions (n = 4). **f**, basal cytosolic fructose bisphosphate (FBP) levels shown as Hylight emission ratio (mean +/-SD) in control and PS1 KD INS-1 cells (n=4). **g**, cellular aspartate levels (mean ± SD) of INS1 cells represented as normalized NMR intensity units (a.u.) under control and PS1 KD conditions (n = 4). **h**, basal cytosolic pyruvate levels shown as Pyronic emission ratio (mean +/-SEM) in control and PS1 KD INS-1 cells with OE of WT and mutant citrin constructs (control: no OE n=67, WT citrin OE n=38, mut1 OE n=38, mut2 OE n=42; PS1 KD: no OE n=88, WT citrin OE n=38, mut1 OE n=45, mut2 OE n=47). Unpaired t-test, n.s. $p > 0.05$, * $p < 0.05$.

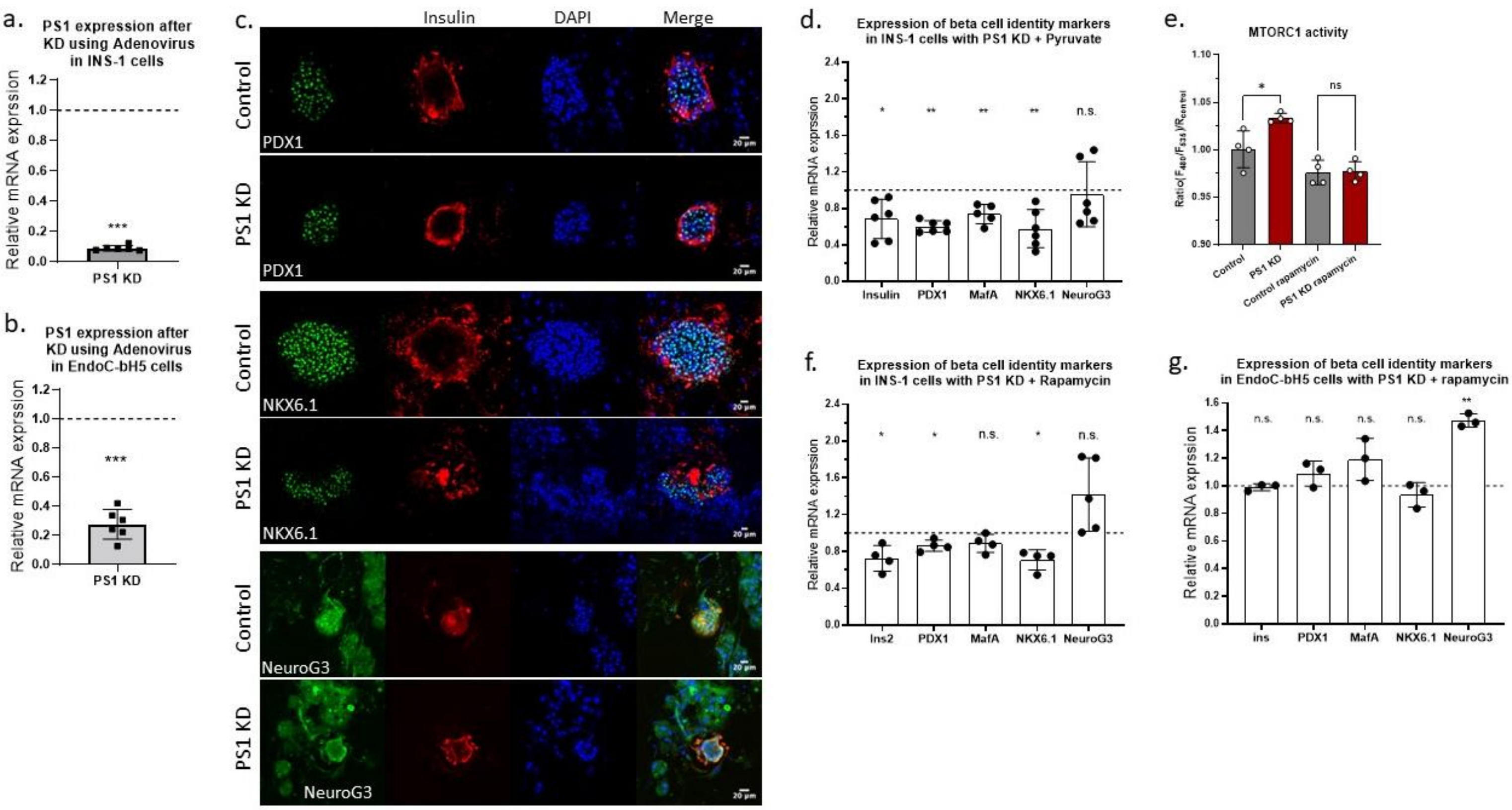

Supplementary figure 4. **a-b,** PS1 KD efficiency in INS-1 cells (**a**, n=6) and EndoC-bH5 (**b**, n=6) cells after using shRNA carrying adenovirus, normalized to control shRNA treated cells. **c**, representative immunofluorescence images of primary pancreatic slices stained with anti-insulin (red), anti-PDX1 (upper panels, green), anti-NKX6.1 (middle panels, green), and anti-Neurogenin 3 (lower panels, green) antibodies, quantified in **Figure 4 c**, scale bar is 20 µm. **d**, bar graphs show mean (+/-SD) mRNA expression of beta cell identity markers in PS1 KD INS1 cells supplemented with pyruvate for 48 hours prior to the analysis; values are normalized to pyruvate treated controls, n=6. **e**, mTORC1 activity measured with TORCAR[58] in INS-1 cells and shown as mean emission ratio (+/- SD), n=4. **f** and **g**, bar graphs show mean (+/-SD) mRNA expression of beta cell identity markers in PS1 KD INS1 (**f**) and EndoC-bH5 (**g**) cells treated with rapamycin, values are normalized to control cells treated with 20 nM rapamycin for 48 hours prior to the analysis, n=3-5. Unpaired t-test (**e**), paired t-test (**a-d, f**), n.s. p > 0.05, * p < 0.05, ** p < 0.01, *** p < 0.001.

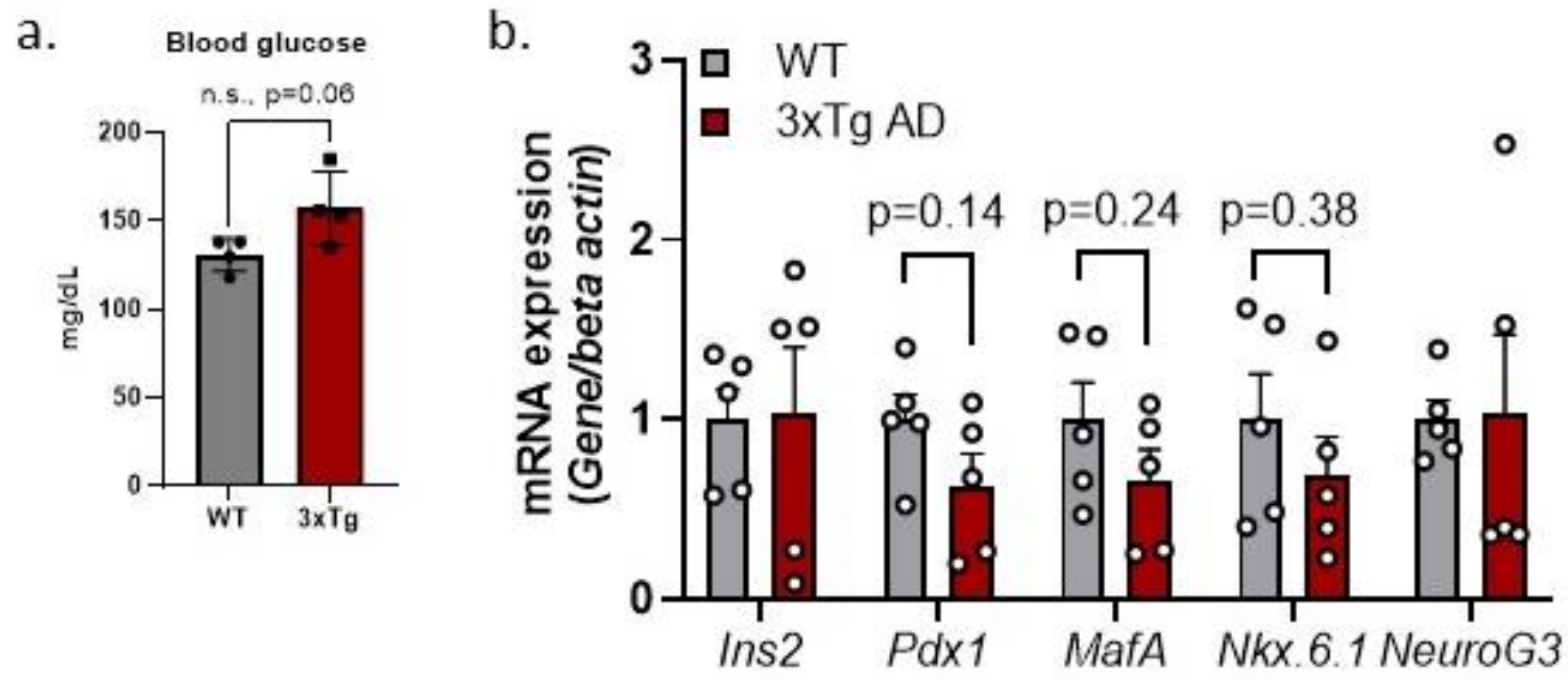

Supplementary figure 5. a, blood glucose level in WT and 3xTg FAD mice, n=4. **b,** expression of beta cell identity markers in pancreatic islets from WT and 3xTg FAD; values are normalized to respective average WT expression (n=5). P values indicated in the figures.
