# Supplementary material for: Presenilin-1 controls glycolysis and identity of pancreatic beta cells
Source: Commun Biol. 2026 Jul 1;9:1315. doi: 10.1038/s42003-026-10581-6 (PMC13649773; doi:10.1038/s42003-026-10581-6)
Supplement: Supplementary file 3 [file 42003_2026_10581_MOESM3_ESM.pdf]

Description of Additional Supplementary Files

File name: Supplementary Data 1

Description: Numerical source data underlying graphs in this article can be found in Supplementary data file.
